# Semi-Synthesis of Harringtonolide Derivatives and Their Antiproliferative Activity

**DOI:** 10.3390/molecules26051380

**Published:** 2021-03-04

**Authors:** Xiutao Wu, Lijie Gong, Chen Chen, Ye Tao, Wuxi Zhou, Lingyi Kong, Jianguang Luo

**Affiliations:** State Key Laboratory of Natural Medicines, Jiangsu Key Laboratory of Bioactive Natural Product Research, School of Traditional Chinese Pharmacy, China Pharmaceutical University, 24 Tong Jia Xiang, Nanjing 210009, China; wxtcpuer@163.com (X.W.); jieligong313@163.com (L.G.); chenchen02544@163.com (C.C.); tycpuer@163.com (Y.T.); zhouwuxi1988@163.com (W.Z.)

**Keywords:** harringtonolide, troponoids, structure-activity relationship (SAR), antiproliferative activity

## Abstract

Harringtonolide (HO), a natural product isolated from *Cephalotaxus harringtonia*, exhibits potent antiproliferative activity. However, little information has been reported on the systematic structure−activity relationship (SAR) of HO derivatives. Modifications on tropone, lactone, and allyl positions of HO (**1**) were carried out to provide 17 derivatives (**2**–**13, 11a**–**11f**). The in vitro antiproliferative activity against four cancer cell lines (HCT-116, A375, A549, and Huh-7) and one normal cell line (L-02) was tested. Amongst these novel derivatives, compound **6** exhibited comparable cell growth inhibitory activity to HO and displayed better selectivity index (SI = 56.5) between Huh-7 and L-02 cells. The SAR results revealed that the tropone and lactone moieties are essential for the cytotoxic activities, which provided useful suggestions for further structural optimization of HO.

## 1. Introduction

**Figure 1:**
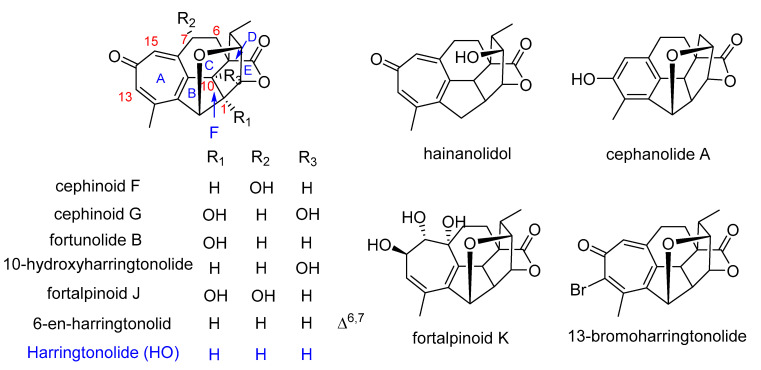
Structures of harringtonolide and its analogues.

Cephalotane-type diterpenoids are widely distributed in the plant of the *Cephalotaxus* genus [1,2,3,4,5], which is the sole member of the Cephalotaxaceae family. Harringtonolide (also named as hainanolide, HO) is the first member of cephalotane-type diterpenoids that was isolated and structurally identified in 1978 from the seeds of *Cephalotaxus harringtonia* by Buta and coworkers. Structurally, HO, as a typical cephalotane-type diterpenoid, has a tropone ring, a fused tetracarbocyclic skeleton (A−B−C−D ring), a bridged lactone (E-ring), and a tetrahydrofuran ring (F-ring) in the molecule. Right after its isolation, it was shown to possess potent plant growth inhibitory [6], antiviral [7], anti-inflammatory [8], and antiproliferative activities [1,8,9]. During the past decades, HO has attracted considerable attention of synthetic chemists because of its unique cage-like troponoid skeleton and remarkable biological effects [10,11,12,13,14,15,16,17,18]. However, great efforts have been mainly devoted to the total synthesis of HO. Therefore, almost all of these HO derivatives (Figure 1) were extracted and separated from the kingdom of plants, such as hainanolidol with F-ring-opening [19], cephanolide A with A-ring-contracted [20], fortalpinoid K without a complete tropone moiety [21], fortalpinoid J [21], cephinoids F-G [8], fortunolide B [22] and 10-hydroxyharringtonolide [23] with hydroxy substitutions in HO, and 6-en-harringtonolide with a double bond adjacent to tropone unit [23]. Only 13-Bromoharringtonolide is the semi-synthetic HO derivative reported by Evanno and coworkers [9]. However, to our knowledge, detailed anti-proliferative structure–activity relationship (SAR) for HO (**1**) is still insufficient up to now. In order to enrich the chemical diversity of HO and discover pharmacologically interesting compounds, a series of novel HO derivatives were semi-synthesized through substitution at tropone, selective reduction of lactone, and allylic oxidation. Moreover, the antiproliferative activity of these derivatives against the HCT-116 (human colon cancer), A375 (human melanoma cancer), A-549 (human lung adenocarcinoma cancer), and Huh-7 (human hepatoma cancer) was evaluated, and their SARs were discussed in the present study.

## 2. Results

### 2.1. Chemical Synthesis

**Scheme 1:**
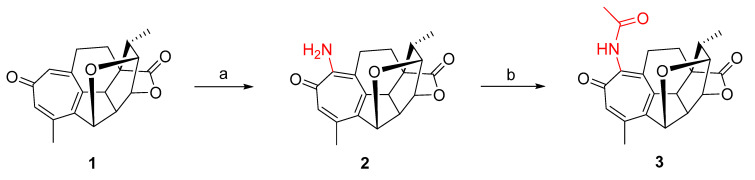
Reagents: (**a**) NH_2_-NH_2_·H_2_O, EtOH and (**b**) AcCOCl, Et_3_N.

Two C-15 substituted analogues (**2** and **3**) were synthesized to probe and define the importance of the C-15 substituent. Scheme 1 outlines the synthesis of target compounds **2** and **3**. The reaction of **1** with NH_2_-NH_2_·H_2_O in EtOH at room temperature (rt) produced **2** in 96% yield. Further amidation of **2** with acetyl chloride and Et_3_N in CH_2_Cl_2_ at rt provided **3** in 92% yield.

**Scheme 2:**
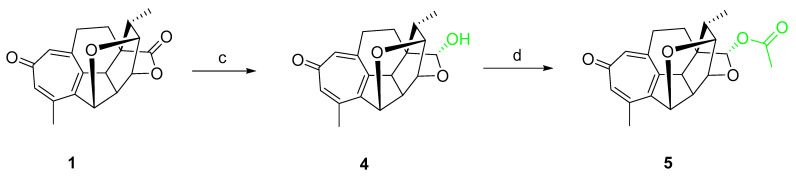
Reagents: (**c**) DIBAL-H, CH_2_Cl_2_ and (**d**) Ac_2_O, Pyr.

To explore the role of lactone, compounds with reduced lactone ring (**4** and **5**) were synthesized as shown in Scheme 2. Selective reduction of the lactone with DIBAL-H at −78 °C afforded lactol **4** in 24% yield, which subsequently underwent an esterification reaction with Ac_2_O to give compound **5** in 92% yield.

**Scheme 3:**
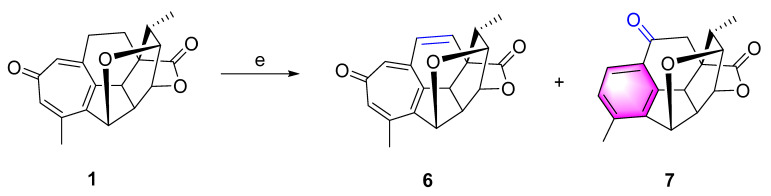
Reagents: (**e**) SeO_2_/TBHP and CH_2_Cl_2_.

In order to discover HO derivatives with diverse allyl alcohol replacements, allylic oxidation of HO with SeO_2_ was conducted. Interestingly, under the condition of Riley oxidation (SeO_2_/TBHP) at rt, compounds **6** with a double bond between C-6 and C-7 in 16% yield and **7** with A-ring contracted in 5% yield were produced as shown in Scheme 3. The structure of compound **6** is consistent with the natural product 6-en-harringtonolide [23] isolated from *Cephalotaxus mannii* as shown in Figure 1. The formation of compound **6** was likely based on the elimination of hydroxyl. A possible mechanism for the formation of **7** is presented in Scheme S1, which is meaningful to explore the conversion of tropone to benzene ring in cephalotane-type diterpenoids.

**Scheme 4:**
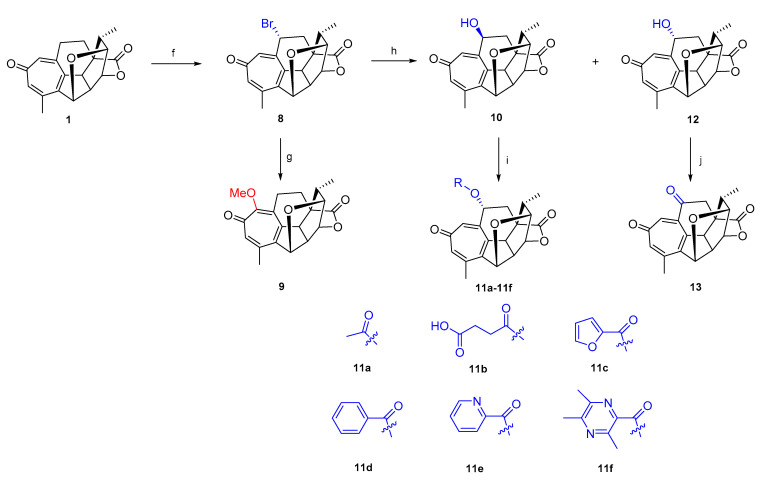
Reagents: (**f**) NBS/AIBN, DCE; (**g**) MeONa/MeOH; (**h**) AgBF_4_, acetone/H_2_O; (**i**) RCOOH, EDCI/DMAP; and (**j**) DMP, CH_2_Cl_2_.

Next, another route was designed to obtain our target compounds. At the beginning, HO (**1**) was treated with NBS/AIBN at 65 °C for 12 h to obtain compound 7*α*-Br harringtonolide (**8**) in 54% yield. Compound **8** was then subjected to hydrolysis with AgBF_4_ in acetone/H_2_O at 65 °C to produce 7*β*-OH harringtonolide (**10**) in 47% yield and 7*α*-OH harringtonolide (**12**) in 21% yield (Scheme 4). The *β*-orientation of 7-OH in compound **10** was deduced from the ROESY correlations (Figure S1) of 7-H with 1-H and 10-H. The structure of compound **12** is identical to the natural product cephinoid F [8] isolated from *Cephalotaxus lanceolata* as shown in Figure 1.

Furthermore, to explore the effects of hydroxy group and its stereochemistry of HO on antiproliferative activity, a series of HO derivatives were designed in which the hydroxy at C-7 was modified as shown in Scheme 4. The cytotoxicity assay (*vide infra*) of these two compounds indicated that the stereochemistry at C-7 influences the cytotoxic activities with *β*-orientation favored. Thus, compound **10** was then coupled with acyl moieties to its 7*β*-OH position to afford a series of new ester derivatives **11a**–**11f**. The 3,5,6-trimethylpyrazine-2-carboxylic acid (TMPA), used in the synthesis of **11f**, was obtained according to the previous report [24]. Interestingly, treatment of **8** with MeONa/MeOH at rt provided **9** with a methoxy substituent at tropone in 72% yield rather than allyl position, the generation of **9** may be through the rearrangement of allyl carbocation and subsequent replacement by methoxy anion (Scheme S2). The oxidation of hydroxy in compound **12** with Dess-Martin periodinane (DMP) in CH_2_Cl_2_ at rt produced **13** in 78% yield.

### 2.2. Cytotoxicity

All the synthesized derivatives of HO were tested for their antiproliferative activity against four human cancer cell lines, including the HCT-116, A375, A-549, and Huh-7 cell lines, using the MTT assay. Cisplatin was used as a positive control, while lead compound HO (**1**) was also included in the study for comparison. IC_50_ values (50% inhibition concentration of cell viability) of the tested compounds were summarized in Table 1.

Parent compound **1** showed strong potency against HCT-116, A375, A549, and Huh-7 cells with IC_50_ values of 0.61, 1.34, 1.67, and 1.25 μM, respectively. Unfortunately, compounds **2**-**5**, **7**, and **9** were inactive against four tested cancer cells at 50 μM. The results revealed that structural variations in the tropone ring and lactone will dramatically impact the cytotoxic activities. 7-OH, 7-keto, or 7-ester group derivatives **10**–**13** showed weak cytotoxic activities or were inactive. In particular, compound **6** showed comparable cytotoxic activities to HO against HCT-116 and Huh-7 cells with IC_50_ values of 0.86 and 1.19 μM, respectively.

The selectivity index is crucial for drug development, because it could mediate side effects, including tissues toxicity [25,26]. Therefore, according to cytotoxic activity results, compounds **6** and **10** with potent cytotoxicity in Huh-7 cells were selected to investigate the selectivity between normal and cancer cells. These two compounds were tested on human normal hepatic L-02 cells with HO as control. The results are listed in Table 2. Interestingly, compound **6** displayed obvious selectivity between Huh-7 and L-02 cells with SI = 56.5 compared with the parent compound **1** with SI = 2.8.

## 3. Discussion

**Figure 2:**
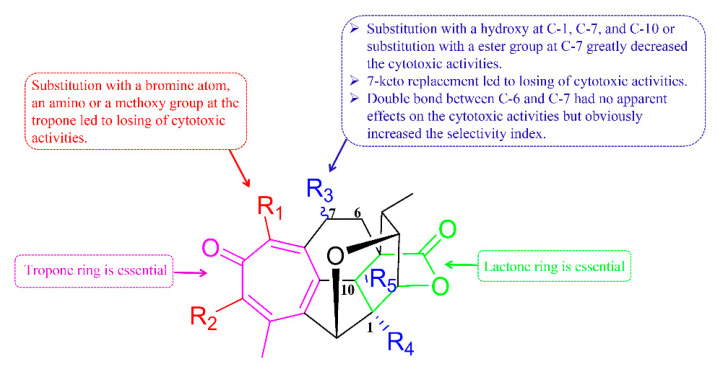
Systematic structure−activity relationship (SAR) of harringtonolide (HO) derivatives.

Combined with previous reports [8,21], systematic SAR of the HO analogues as antiproliferative agents (Figure 2) was discussed as follows based on above-described results. (1) From the screening results in Table 1, it was observed that substitution with a bromine atom, an amino or a methoxy group at tropone (2-bromoharringtonolide [9] and compounds **2–3**, **9**), lack of tropone ring (fortalpinoid K [21]), and A-ring contraction of tropone (cephanolide A [20] and compound **7**) in HO led to losing of cytotoxic activities. (2) Reduction of lactone in HO also led to losing of cytotoxic activities as observed in the compounds **4** and **5**. These results indicated that the tropone and lactone moieties are essential for the cytotoxic activities. (3) The introduction of an *α*-oriented hydroxyl at C-7 or other positions drastically decreased cytotoxic activities in accord with previous reports [8,21,22,23], whereas the presence of a 7*β*-OH exerts no obvious effects on the cytotoxic activities against HCT-116 cells as observed in the cases of compounds **10** (IC_50_ = 2.29 μM) and **12** (IC_50_ = 31.88 μM). However, further esterification at 7*β*-OH greatly decreased cytotoxic activities against A549 cells in different levels as observed in compounds **11a** (IC_50_ = 27.49 μM), **11c** (IC_50_ = 23.25 μM), **11e** (IC_50_ = 17.98 μM), and **11f** (IC_50_ = 25.95 μM) compared with HO (IC_50_ = 1.67 μM) or led to losing of cytotoxic activities as observed in compounds **11b** and **11d** (IC_50_ > 50 μM against tested cells). The compounds **11a**, **11c**, **11e**, and **11f** also showed weak cytotoxic activities against three other cancer cell lines. This could be attributed to the steric hindrance that affecting the binding of compounds to their targets [27,28]. (4) The presence of a double bond adjacent to the tropone unit had no obvious effects on the cytotoxic activities against HCT-116 and Huh-7 cells as observed in the cases of compound **6** (IC_50_ = 0.86 and 1.19 μM, respectively) compared with HO (IC_50_ = 0.61 and 1.25 μM, respectively) but obviously increased the selectivity between Huh-7 and L-02 cells, as observed in Table 2. The selectivity index of compound **6** (SI = 56.5) was 20 times higher than HO (SI = 2.8). (5) Carbonyl adjacent to the tropone motif led to losing of cytotoxic activities, as observed in **13** (IC_50_ >50 μM against tested cells).

According to the SAR analysis of HO derivatives, compound **6** exhibited the most potent antiproliferative activity and low toxicity. Our studies indicated that analogue **6** could be further investigated as an antitumor drug candidate. Additionally, there is an urgent need for thorough research to understand the mechanism of action and targets of this kind of analogues.

## 4. Materials and Methods

### 4.1. Chemistry

HO, used as starting material, was isolated from *Cephalotaxus fortunei* Hook. f. by our group, and its structure was identified by ESI-MS and NMR. All reagents were purchased from Energy Chemical (Shanghai, China) and Aladdin (Shanghai, China) and were used without any further purification. Thin-layer chromatography (TLC) was performed using silica gel plates (GF254, Qingdao Marine Chemical Ltd., Qingdao, China) and visualized under ultraviolet (UV) light (254 nm). Silica gel column chromatography was performed using 200–300 mesh (Qingdao Marine Chemical Ltd., Qingdao, China).

NMR spectra were recorded on Bruker Avance III-500 and Bruker Avance III-600 spectrometers (Bruker, Karlsruhe, Germany) at ambient temperature using TMS as the internal standard. High-resolution electrospray ionization (HRESI) mass spectra were carried out using an Agilent 6520B Q-TOF mass spectrometer (Agilent Technologies, Santa Clara, CA, USA). All final compounds have purity >95% (Figure S2) as determined by an Agilent 1260 instrument equipped with VWD detector (Agilent Technologies, Santa Clara, CA, USA) using a COSMOSIL 5C18-AR-II column (250 mm × 4.6 mm, i.d., 5 μm, Nacalai Tesque, Kyoto, Japan).

#### 4.1.1. General Procedure for the Synthesis of Compounds **2** and **3**

To a solution of **1** (310 mg, 1 mmol) in EtOH (10 mL), 80% hydrazine monohydrate (NH_2_-NH_2_·H_2_O; 5 mL) was added. The solution was allowed to stir at rt for 8 h until all starting material was consumed as determined by TLC. The reaction was quenched by the addition of H_2_O, and the mixture was extracted with CH_2_Cl_2_. The combined organic layers were dried over Na_2_SO_4_, and concentrated under reduced pressure. The residue was purified by column chromatography using CH_2_Cl_2_-MeOH (10:1) to afford **2** as a yellow solid (312 mg, 96%).

*(3R,4aR,10aS,11S)-8-amino-5,11-dimethyl-2a,3,4a,9,10,10a1-hexahydro-1H-2,4-dioxa-3,10a-methanocyclohepta[bc]cyclopenta[jk]acenaphthylene-1,7(2a1H)-dione (**2**).*

^1^H-NMR (600 MHz, CDCl_3_): *δ* 7.18 (s, 1H), 6.07 (brs, 2H), 5.43 (d, *J* = 4.9 Hz, 1H), 5.19 (t, *J* = 5.5 Hz, 1H), 3.95 (d, *J* = 5.7 Hz, 1H), 3.38-3.32 (m, 2H), 2.90 (ddd, *J* = 14.6, 7.8, 1.3 Hz, 1H), 2.80 (ddd, *J* = 15.5, 6.4, 1.4 Hz, 1H), 2.65 (ddd, *J* = 15.4, 11.9, 7.7 Hz, 1H), 2.42 (s, 3H), 1.68 (q, *J* = 7.6 Hz, 1H), 1.29 (ddd, *J* = 14.6, 11.9, 6.4 Hz, 1H), 0.86 (d, *J* = 7.6 Hz, 3H). ^13^C-NMR (151 MHz, CDCl_3_): *δ* 175.67, 174.19, 152.80, 148.32, 144.17, 135.71, 132.71, 118.29, 85.95, 80.21, 78.99, 50.79, 45.47, 42.01, 40.56, 25.35, 24.00, 22.91, 14.81. HR-MS (ESI) *m/z*: 443.1101 [M + Na]^+^ (Calcd for C_19_H_19_NNaO_4_: 443.1101).

To a solution of **2** (8.1 mg, 0.025 mmol) in 1 mL of anhydrous dichloromethane, triethylamine (TEA; 6.9 μL, 0.05 mmol) was added, and then, acetyl chloride (2.7 μL, 0.0375 mmol) was added to the solution at 0 °C. The resulting solution was allowed to warm to rt and stirred for 4 h. Then, the reaction was quenched with water and extracted with dichloromethane. The combined organic phases were washed with brine, dried over Na_2_SO_4_, and concentrated in vacuo. The residue was purified by column chromatography using hexanes-EtOAc (1:2) to afford **3** as a yellow solid (8.4 mg, 92%).

*N-((3R,4aR,10aS,11S)-5,11-dimethyl-1,7-dioxo-2a,2a1,3,4a,7,9,10,10a1-octahydro-1H-2,4-dioxa-3,10a-methanocyclohepta[bc]cyclopenta[jk]acenaphthylen-8-yl) acetamide (**3**).*

^1^H-NMR (500 MHz, CDCl_3_): *δ* 8.68 (s, 1H), 7.22 (s, 1H), 5.40 (d, *J* = 5.5 Hz, 1H), 5.20 (t, *J* = 5.7 Hz, 1H), 3.97 (d, *J* = 5.6 Hz, 1H), 3.52 (d, *J* = 8.9 Hz, 1H), 3.39 (dt, *J* = 8.9, 5.7, 1H), 2.80 (dq, *J* = 12.7, 8.0 Hz, 2H), 2.64-2.57 (m, 1H), 2.43 (s, 3H), 2.26 (s, 3H), 1.70 (q, *J* = 7.6 Hz, 1H), 1.21-1.13 (m, 1H), 0.86 (d, *J* = 7.5 Hz, 3H). ^13^C-NMR (126 MHz, CDCl_3_): *δ* 179.73, 173.72, 168.74, 147.10, 145.28, 143.03, 141.91, 138.19, 136.84, 86.21, 80.24, 79.72, 50.89, 45.64, 42.26, 40.30, 30.29, 24.58, 24.15, 22.31, 14.87. HR-MS (ESI) *m/z*: 390.1311 [M + Na]^+^ (Calcd for C_21_H_21_NNaO_5_: 390.1312).

#### 4.1.2. General Procedure for the Synthesis of Compounds **4** and **5**

To a solution of **1** (155 mg, 0.5 mmol) in CH_2_Cl_2_ (5 mL), diisobutylaluminum hydride (DIBAL-H; 1.0 M solution in hexane, 1.3 mL, 1.3 mmol) was added in 3 portions at −78 °C under nitrogen, and the mixture was stirred at −78 °C for 3 h. Saturated aqueous sodium potassium tartrate was then added to quench the reaction. The resulting mixture was extracted with CH_2_Cl_2_. The combined organic layers were washed with brine, dried over anhydrous Na_2_SO_4_, filtered, and concentrated under reduced pressure. The residue was purified by column chromatography on silica gel by eluting with CH_2_Cl_2_-MeOH (20:1) to furnish the corresponding lactol **4** as a yellow solid (37.5 mg, 24%).

*(1R,3R,4aR,10aS,11S)-1-hydroxy-5,11-dimethyl-2a,3,4a,9,10,10a1-hexahydro-1H-2,4-dioxa-3,10a-methanocyclohepta[bc]cyclopenta[jk]acenaphthylen-7(2a1H)-one (**4**).*

^1^H-NMR (600 MHz, CDCl_3_): *δ* 6.98 (s, 1H), 6.92 (s, 1H), 5.33 (d, *J* = 5.3 Hz, 1H), 5.15 (s, 1H), 4.61 (t, *J* = 5.7 Hz, 1H), 3.72 (d, *J* = 5.7 Hz, 1H), 3.69(d, *J* = 8.7 Hz, 1H), 3.08 (dt, *J* = 8.8, 5.5 Hz, 1H), 2.91-2.83 (m, 1H), 2.57 (dd, *J* = 14.9, 5.9 Hz, 1H), 2.38 (s, 3H), 2.05 (dd, *J* = 13.9, 8.1 Hz, 1H), 1.40 (q, *J* = 7.7 Hz, 1H), 1.30 (td, *J* = 13.1, 5.9 Hz, 2H), 0.91 (d, *J* = 7.6 Hz, 3H). ^13^C-NMR (151 MHz, CDCl_3_): *δ* 186.78, 149.61, 146.69, 146.50, 144.64, 140.76, 138.95, 91.82, 87.42, 80.99, 74.86, 42.68, 41.05, 39.54, 38.67, 33.14, 24.79, 24.30, 13.72. HR-MS(ESI) *m/z*: 335.1252, [M + Na]^+^ (Calcd for C_19_H_20_NNaO_4_: 335.1254).

To a solution of **4** (10 mg, 0.032 mmol) in dry pyridine (0.5 mL, 6.2 mmol) at 0 °C, acetic anhydride (0.5 mL, 5.3 mmol) was added slowly. The reaction mixture was then allowed to warm to room temperature and stirred for 2 h. The mixture was slowly poured into 15 mL of fast-stirring ice water. The solution was extracted with CH_2_Cl_2_ and washed with 1 M HCl, water, and brine. After the solution was dried over Na_2_SO_4_ and filtered, the solvent was removed to give a crude product. The residue was purified by column chromatography on silica gel by eluting with hexanes-EtOAc (1:1) to furnish the corresponding **5** as a yellow solid (10.4 mg, 92%).

*(1S,3R,4aR,10aS,11S)-5,11-dimethyl-7-oxo-2a,2a1,3,4a,7,9,10,10a1-octahydro-1H-2,4-dioxa-3,10a-methanocyclohepta[bc]cyclopenta[jk]acenaphthylen-1-yl acetate (**5**).*

^1^H-NMR (500 MHz, CDCl_3_): *δ* 6.95 (s, 1H), 6.87 (s, 1H), 6.01 (s, 1H), 5.33 (d, *J* = 5.4 Hz, 1H), 4.66 (t, *J* = 5.7 Hz, 1H), 3.75 (d, *J* = 5.6 Hz, 1H), 3.61(d, *J* = 8.8 Hz, 1H), 3.10 (dt, *J* = 8.7, 5.5 Hz, 1H), 2.82 (dddd, *J* = 14.6, 12.3, 8.1, 2.0 Hz, 1H), 2.56 (dd, *J* = 15.0, 6.0 Hz, 1H), 2.36 (s, 3H), 2.18 (s, 3H), 1.83 (dd, *J* = 14.2, 8.0 Hz, 1H), 1.49 (q, *J* = 7.6 Hz, 1H), 1.37 (ddd, *J* = 14.2, 12.4, 6.1 Hz, 1H), 0.97 (d, *J* = 7.6 Hz, 3H). ^13^C-NMR (126 MHz, CDCl_3_): *δ* 186.76, 170.79, 148.24, 146.30, 145.77, 144.28, 141.03, 139.15, 91.92, 87.46, 80.72, 75.18, 43.77, 41.07, 38.54, 38.28, 32.90, 24.38, 24.20, 21.61, 13.67. HR-MS(ESI) *m/z*: 377.1354, [M + Na]^+^ (Calcd for C_21_H_22_NaO_5_: 377.1359).

#### 4.1.3. General Procedure for the Synthesis of Compounds **6** and **7**

A round-bottom flask was charged with selenium dioxide (SeO_2_, 14.2 mg, 0.128 mmol) and tert-butyl hydroperoxide (TBHP, 5.5 M solution in hexane, 145.5 μL, 0.8 mmol). The resulting mixture was diluted with dichloromethane (4.0 mL) and stirred at rt for 30 min. Then, the solution was added to compound **1** (100 mg, 0.32 mmol) in CH_2_Cl_2_ (2 mL) and stirred for the next 24 h. The reaction was quenched with saturated aqueous Na_2_SO_3_ solution and extracted with CH_2_Cl_2_. The combined organic layers were washed with brine and dried (Na_2_SO_4_), the solvent was removed to give a crude product. The obtained crude product was purified by column chromatography hexanes-EtOAc (3:2) to afford **6** (15.8 mg, 16%) and **7** (4.7 mg, 5%) as a yellow solid.

*(3R,4aR,10aS,11S)-5,11-dimethyl-2a,3,4a,10a1-tetrahydro-1H-2,4-dioxa-3,10a methanocyclohepta[bc]cyclopenta[jk]acenaphthylene-1,7(2a1H)-dione (**6**).*

^1^H-NMR (600 MHz, CDCl_3_): *δ* 6.80 (s, 1H), 6.69 (d, *J* = 9.5 Hz, 1H), 6.61 (d, *J* = 2.5 Hz, 1H), 6.42 (d, *J* = 9.6 Hz, 1H), 5.34 (d, *J* = 5.1 Hz, 1H), 5.23(t, *J* = 5.7 Hz, 1H), 4.03 (d, *J* = 5.6 Hz, 1H), 3.61 (d, *J* = 8.9 Hz, 1H), 3.34 (dt, *J* = 9.0, 5.4 Hz, 1H), 2.32 (s, 3H), 2.20 (q, *J* = 7.6 Hz, 1H), 0.95 (d, *J* = 7.6 Hz, 3H). ^13^C-NMR (151 MHz, CDCl_3_): *δ* 187.69, 171.24, 144.65, 143.89, 142.98, 140.33, 139.29, 136.17, 134.28, 131.23, 86.53, 80.58, 80.35, 52.28, 47.99, 41.56, 39.99, 23.48, 15.95. HR-MS(ESI) *m/z*: 331.0941, [M + Na]^+^ (Calcd for C_19_H_16_NaO_4_: 331.0941).

*(3R,4aR,9aS,10S)-5,10-dimethyl-2a,2a1,4a,9a1-tetrahydro-1H,3H-2,4-dioxa-3,9a-methanopentaleno [4,3,2,1-cdef]phenanthrene-1,8(9H)-dione (**7**).*

^1^H-NMR (600 MHz, CDCl_3_): *δ* 7.65 (d, *J* = 7.7 Hz, 1H), 7.28 (s, 1H), 5.60 (d, *J* = 4.1 Hz, 1H), 5.25 (t, *J* = 5.3 Hz, 1H), 3.88 (d, *J* = 5.6 Hz, 1H), 3.65-3.58 (m, 2H), 3.08 (d, *J* = 19.3 Hz, 1H), 2.68 (d, *J* = 19.3 Hz, 1H), 2.48 (s, 3H), 1.21 (q, *J* = 7.6 Hz, 1H), 0.83 (d, *J* = 7.6 Hz, 3H). ^13^C-NMR (151 MHz, CDCl_3_): *δ* 194.75, 172.67, 147.35, 143.87, 141.41, 131.60, 129.08, 127.23, 82.94, 80.08, 78.94, 47.56, 47.13, 42.81, 40.14, 37.99, 18.91, 15.29. HR-MS(ESI) *m/z*: 319.0939, [M + Na]^+^ (Calcd for C_18_H_16_NaO_4_: 319.0941).

#### 4.1.4. General Procedure for the Synthesis of Compounds **8**–**13**

To a stirred solution of **1** (620 mg, 2 mmol) in 1,2-dichloroethane (100 mL), N-Bromosuccinimide (NBS; 427.2 mg, 2.4 mmol), in 3 portions every 6 h, and azodiisobutyronitrile (AIBN; 10.7 mg, 0.065 mmol) were added. The reaction mixture was heated at 80 °C for 18 h. After cooling, the reaction solution was evaporated in vacuo and diluted with CH_2_Cl_2_, washed with H_2_O and brine, dried, and concentrated. The residue was purified by column chromatography on silica gel CH_2_Cl_2_-EtOAc (20:1) to give **8** as a yellow solid (419 mg, 54%).

*(3R,4aR,9R,10aS,11S)-9-bromo-5,11-dimethyl-2a,3,4a,9,10,10a1-hexahydro-1H-2,4-dioxa-3,10a-methanocyclohepta[bc]cyclopenta[jk]acenaphthylene-1,7(2a1H)-dione (**8**).*

^1^H-NMR (500 MHz, CDCl_3_): *δ* 6.97 (s, 1H), 6.94 (d, *J* = 2.5 Hz, 1H), 5.37 (d, *J* = 5.3 Hz, 1H), 5.23 (t, *J* = 5.7 Hz, 1H), 5.16 (d, *J* = 6.2 Hz, 1H), 4.46 (d, *J* = 9.0 Hz, 1H), 4.01 (d, *J* = 5.6 Hz, 1H), 3.47 (dt, *J* = 8.9, 5.6 Hz, 1H), 3.34 (d, *J* = 17.0 Hz, 1H), 2.38 (s, 3H), 2.11 (dd, *J* = 17.0, 6.2 Hz, 1H), 1.79 (q, *J* = 7.6 Hz, 1H), 0.87 (d, *J* = 7.5 Hz, 3H). ^13^C-NMR (126 MHz, CDCl_3_): *δ* 186.28, 172.59, 147.43, 144.36, 144.19, 143.14, 141.90, 139.12, 85.81, 80.00, 79.78, 47.82, 47.23, 45.84, 41.67, 40.04, 32.78, 24.22, 14.88. HR-MS(ESI) *m/z*: 411.0199, [M + Na]^+^ (Calcd for C_19_H_17_BrNaO_4_: 411.0202).

To a solution of **8** (10 mg, 0.026 mmol) in MeOH (0.5 mL) at 0 °C, MeONa (3.5 mg, 0.064 mmol) was added slowly. The reaction mixture was then allowed to warm to room temperature and stirred for 3 h. The mixture was slowly poured into 10 mL water to quench the reaction. The solution was extracted with CH_2_Cl_2_ and washed with water, and brine. After the solution was dried over Na_2_SO_4_ and filtered, the solvent was removed to give a crude product. The residue was purified by column chromatography on silica gel by eluting with hexanes-EtOAc (3:2) to furnish the corresponding **9** as a white solid (7.8 mg, 88%).

*(3R,4aR,10aS,11S)-8-methoxy-5,11-dimethyl-2a,3,4a,9,10,10a1-hexahydro-1H-2,4-dioxa-3,10a-methanocyclohepta[bc]cyclopenta[jk]acenaphthylene-1,7(2a1H)-dione (**9**).*

^1^H-NMR (600 MHz, CDCl_3_): *δ* 7.04 (s, 1H), 5.36 (d, *J* = 5.2 Hz, 1H), 5.19 (t, *J* = 5.6 Hz, 1H), 3.97 (d, *J* = 5.6 Hz, 1H), 3.92(s, 3H), 3.45 (ddd, *J* = 15.9, 6.4, 1.3, 1H), 3.40-3.33 (m, 2H), 2.84 (ddd, *J* = 14.6, 8.1, 1.3, 1H), 2.46 (ddd, *J* = 15.9, 12.3, 8.1 Hz, 1H), 2.37 (s, 3H), 1.74 (q, *J* = 7.6 Hz, 1H), 1.21 (ddd, *J* = 14.7, 12.4, 6.4 Hz, 1H), 0.88 (d, *J* = 7.6 Hz, 3H). ^13^C-NMR (151 MHz, CDCl_3_): *δ* 180.89, 173.87, 162.43, 145.58, 143.26, 141.63, 138.65, 132.38, 85.83, 80.03, 79.51, 59.19, 50.30, 46.04, 41.91, 40.59, 24.51, 23.34, 22.32, 14.83. HR-MS(ESI) *m/z*: 363.1202, [M + Na]^+^ (Calcd for C_20_H_20_NaO_5_: 363.1203).

Compound **8** (388 mg, 1 mmol) was dissolved in a solution of acetone and water (1:1, 5 mL) and then AgBF_4_ (973.5 mg, 5 mmol) was added. After stirring for 2 h at 70 °C, the reaction mixture was diluted with CH_2_Cl_2_ and washed with water. The layers were separated and the aqueous layer was extracted with CH_2_Cl_2_. The combined organic layers were dried over Na_2_SO_4_, filtered, and concentrated under reduced pressure. The crude residue was purified by column chromatography hexanes-EtOAc (1:2) to afford **10** (153.2 mg, 47% yield) and **12** as yellow solids (68.5 mg, 21% yield).

*(3R,4aR,9S,10aS,11S)-9-hydroxy-5,11-dimethyl-2a,3,4a,9,10,10a1-hexahydro-1H-2,4-dioxa-3,10a-methanocyclohepta[bc]cyclopenta[jk]acenaphthylene-1,7(2a1H)-dione (**10**).*

^1^H-NMR (600 MHz, CDCl_3_): *δ* 7.41 (s, 1H), 7.00 (s, 1H), 5.35 (d, *J* = 5.4 Hz, 1H), 5.18 (t, *J* = 5.7 Hz, 1H), 4.87 (t, *J* = 9.1 Hz, 1H), 3.97 (d, *J* = 5.6 Hz, 1H), 3.45 (d, *J* = 8.9 Hz, 1H), 3.36 (dt, *J* = 8.8, 5.7 Hz, 1H), 3.21 (dd, *J* = 14.2, 7.9 Hz, 1H), 2.38 (s, 3H), 1.73 (q, *J* = 7.6 Hz, 1H), 1.18 (dd, *J* = 14.2, 10.7 Hz, 1H), 0.88 (d, *J* = 7.6 Hz, 3H). ^13^C-NMR (151 MHz, CDCl_3_): *δ* 187.20, 173.10, 148.51, 146.34, 144.66, 143.94, 141.81, 135.35, 85.79, 80.10, 79.51, 68.25, 48.44, 44.91, 42.12, 40.09, 31.96, 24.19, 14.86. HR-MS(ESI) *m/z*: 327.1229, [M + H]^+^ (Calcd for C_19_H_19_O_5_: 327.1227).

*(3R,4aR,9R,10aS,11S)-9-hydroxy-5,11-dimethyl-2a,3,4a,9,10,10a1-hexahydro-1H-2,4-dioxa-3,10a-methanocyclohepta[bc]cyclopenta[jk]acenaphthylene-1,7(2a1H)-dione (**12**).*

^1^H-NMR (600 MHz, MeOD): *δ* 6.82 (s, 1H), 6.75 (d, *J* = 2.5 Hz, 1H), 5.78 (d, *J* = 2.6 Hz, 1H), 5.34 (d, *J* = 5.4 Hz, 1H), 5.21 (t, *J* = 5.7 Hz, 1H), 4.61-4.59 (m, 1H), 4.05 (d, *J* = 9.0 Hz, 1H), 3.92 (d, *J* = 5.6 Hz, 1H), 3.40 (dt, *J* = 9.1, 5.6 Hz, 1H), 2.52 (dd, *J* = 15.4, 1.2 Hz, 1H), 2.29 (s, 3H), 1.56–1.46 (m, 2H), 0.70 (d, *J* = 7.5 Hz, 3H). ^13^C-NMR (151 MHz, MeOD): *δ* 195.28, 182.96, 156.04, 155.08, 154.31, 154.02, 149.58, 148.23, 94.68, 89.02, 88.07, 81.60, 56.58, 54.84, 50.35, 48.56, 40.35, 32.95, 24.25. HR-MS(ESI) *m/z*: 327.1228, [M + H]^+^ (Calcd for C_19_H_19_O_5_: 327.1227).

To a solution of compound **10** (10 mg, 0.03 mmol) in 2 mL of anhydrous dichloromethane, 1-(3-(dimethylamino)propyl)-3-ethylcarbodiimide hydrochloride (EDCI; 11.5 mg, 0.06 mmol), DMAP (catalytic amount), and carboxylic acid (0.045 mmol) were added. The resulting solution was stirred at room temperature for 4 h. Then, the reaction was quenched with water and extracted with EtOAc. The combined organic phases were washed with brine, dried over Na_2_SO_4_, and concentrated in vacuo. The residue was subjected to column chromatography hexanes-EtOAc (1:1) to afford **11a**-**11f** as white solids.

*(3R,4aR,9S,10aS,11S)-5,11-dimethyl-1,7-dioxo-2a,2a1,3,4a,7,9,10,10a1-octahydro-1H-2,4-dioxa-3,10a-methanocyclohepta[bc]cyclopenta[jk]acenaphthylen-9-yl acetate (**11a**).* Yield: 96% (10.6 mg).

^1^H-NMR (500MHz, CDCl_3_): *δ* 6.99 (s, 1H), 6.94 (t, *J* = 2.3 Hz, 1H), 5.93 (ddd, *J* = 10.4, 8.0, 2.0 Hz, 1H), 5.36 (d, *J* = 5.4 Hz, 1H), 5.19 (t, *J* = 5.7 Hz, 1H), 3.98 (d, *J* = 5.6 Hz, 1H), 3.52 (d, *J* = 8.8 Hz, 1H), 3.38 (dt, *J* = 9.0, 5.7 Hz, 1H), 3.20 (dd, *J* = 8.8, 5.7 Hz, 1H), 2.38 (s, 3H), 2.22 (s, 3H), 1.79 (q, *J* = 7.5 Hz, 1H), 1.23 (dd, *J* = 14.0, 10.7 Hz, 1H), 0.99 (d, *J* = 7.5 Hz, 3H). ^13^C-NMR (126 MHz, CDCl_3_): *δ* 186.30, 172.31, 169.39, 146.58, 143.89, 142.89, 142.67, 142.18, 135.17, 85.90, 80.04, 79.51, 68.63, 48.74, 44.68, 42.11, 39.97, 29.19, 24.09, 20.98, 14.84. HR-MS(ESI) *m/z*: 391.1145, [M + H]^+^ (Calcd for C_21_H_20_NaO_6_: 391.1152).

*4-(((3R,4aR,9S,10aS,11S)-5,11-dimethyl-1,7-dioxo-2a,2a1,3,4a,7,9,10,10a1-octahydro-1H-2,4-dioxa-3,10a-methanocyclohepta[bc]cyclopenta[jk]acenaphthylen-9-yl)oxy)-4-oxobutanoic acid (**11b**).* Yield: 86% (11.0 mg).

^1^H-NMR (500 MHz, CDCl_3_): *δ* 7.19 (d, *J* = 2.2 Hz, 1H), 7.01 (s, 1H), 5.98 (ddd, *J* = 10.3, 8.1, 1.9 Hz, 1H), 5.37 (d, *J* = 5.4 Hz, 1H), 5.20 (t, *J* = 5.8 Hz, 1H), 3.98 (d, *J* = 5.6 Hz, 1H), 3.55 (d, *J* = 8.8 Hz, 1H), 3.42 (dt, *J* = 8.8, 5.6 Hz, 1H), 3.18 (dd, *J* = 14.0, 8.1 Hz, 1H), 2.91 (ddd, *J* = 16.5, 9.8, 3.2 Hz, 1H), 2.82-2.68 (m, 3H), 2.39 (s, 3H), 1.74 (q, *J* = 7.6 Hz, 1H), 0.88 (d, *J* = 7.5 Hz, 3H). ^13^C-NMR (126 MHz, CDCl_3_): *δ* 186.70, 174.57, 172.35, 171.14, 146.99, 145.03, 143.85, 143.25, 141.93, 135.00, 85.78, 79.95, 79.41, 68.90, 48.61, 44.51, 41.98, 39.80, 29.21, 28.92, 28.81, 24.07, 14.69. HR-MS(ESI) *m/z*: 449.1207, [M + Na]^+^ (Calcd for C_23_H_22_NaO_8_: 449.1207).

*(3R,4aR,9S,10aS,11S)-5,11-dimethyl-1,7-dioxo-2a,2a1,3,4a,7,9,10,10a1-octahydro-1H-2,4-dioxa-3,10a-methanocyclohepta[bc]cyclopenta[jk]acenaphthylen-9-yl 4-morpholino-4-oxobutanoate (**11c**).* Yield: 88% (11.1 mg).

^1^H-NMR (600 MHz, CDCl_3_): *δ* 7.65 (dd, *J* = 1.7, 0.8 Hz, 1H), 7.34 (dd, *J* = 3.5, 0.8 Hz, 1H), 7.05 (t, *J* = 2.2 Hz, 1H), 7.00 (s, 1H), 6.58 (dd, *J* = 3.5, 1.7 Hz, 1H), 6.16 (ddd, *J* = 10.4, 8.1, 2.0 Hz, 1H), 5.38 (d, *J* = 5.3 Hz, 1H), 5.21 (t, *J* = 5.7 Hz, 1H), 4.00 (d, *J* = 5.6 Hz, 1H), 3.58 (d, *J* = 8.7 Hz, 1H), 3.41 (dt, *J* = 8.8, 5.6 Hz, 1H), 3.31 (dd, *J* = 14.0, 8.1 Hz, 1H), 2.39 (s, 3H), 1.84 (q, *J* = 7.6 Hz, 1H), 1.39 (dd, *J* = 14.1, 10.9 Hz, 1H), 0.91 (d, *J* = 7.6 Hz, 3H). ^13^C-NMR (151 MHz, CDCl_3_): *δ* 186.29, 172.26, 156.93, 147.45, 146.67, 143.90, 143.48, 142.72, 142.55, 142.22, 135.33, 119.83, 112.43, 85.90, 80.03, 79.49, 68.86, 48.77, 44.69, 42.12, 39.98, 29.24, 24.11, 14.87. HR-MS(ESI) *m/z*: 443.1101, [M + Na]^+^ (Calcd for C_24_H_20_NaO_7_: 443.1101).

*(3R,4aR,9S,10aS,11S)-5,11-dimethyl-1,7-dioxo-2a,2a1,3,4a,7,9,10,10a1-octahydro-1H-2,4-dioxa-3,10a-methanocyclohepta[bc]cyclopenta[jk]acenaphthylen-9-yl benzoate (**11d**).* Yield: 82% (10.6 mg).

^1^H-NMR (600 MHz, CDCl_3_): *δ* 8.12 (d, *J* = 7.8 Hz, 2H), 7.63 (t, *J* = 7.4 Hz, 1H), 7.49 (t, *J* = 7.6 Hz, 2H), 7.05 (d, *J* = 2.0 Hz, 1H), 7.01 (s, 1H), 6.20 (ddd, *J* = 10.3, 8.1, 1.8 Hz, 1H), 5.39 (d, *J* = 5.3 Hz, 1H), 5.22 (t, *J* = 5.7 Hz, 1H), 4.01 (d, *J* = 5.6 Hz, 1H), 3.61 (d, *J* = 8.6 Hz, 1H), 3.42 (dt, *J* = 9.0, 5.7 Hz, 1H), 3.34 (dd, *J* = 14.0, 8.0 Hz, 1H), 2.39 (s, 3H), 1.86 (q, *J* = 7.5 Hz, 1H), 1.39 (dd, *J* = 14.0, 10.8 Hz, 1H), 0.92 (d, *J* = 7.5 Hz, 3H). ^13^C-NMR (151 MHz, CDCl_3_): *δ* 186.31, 172.34, 164.98, 146.66, 143.90, 143.13, 142.72, 142.22, 135.31, 134.00, 130.09, 128.93, 128.81, 85.93, 80.06, 79.51, 69.08, 48.80, 44.71, 42.17, 40.00, 29.30, 24.12, 14.89. HR-MS(ESI) *m/z*: 453.1308, [M + Na]^+^ (Calcd for C_26_H_22_NaO_6_: 453.1309).

*(3R,4aR,9S,10aS,11S)-5,11-dimethyl-1,7-dioxo-2a,2a1,3,4a,7,9,10,10a1-octahydro-1H-2,4-dioxa-3,10a-methanocyclohepta[bc]cyclopenta[jk]acenaphthylen-9-yl picolinate (**11e**).* Yield: 91% (11.8 mg).

^1^H-NMR (600 MHz, CDCl_3_): *δ* 8.82 (d, *J* = 4.3 Hz, 1H), 8.23 (d, *J* = 7.8 Hz, 1H), 7.91 (d, *J* = 7.5 Hz, 1H), 7.56 (dd, *J* = 7.7, 4.6 Hz, 1H), 7.10 (d, *J* = 2.2 Hz, 1H), 6.99 (s, 1H), 6.29 (ddd, *J* = 10.6, 8.2, 1.9 Hz, 1H), 5.38 (d, *J* = 5.4 Hz, 1H), 5.22 (t, *J* = 5.7 Hz, 1H), 4.01 (d, *J* = 5.6 Hz, 1H), 3.61 (d, *J* = 8.8 Hz, 1H), 3.42 (dt, *J* = 8.8, 5.7 Hz, 1H), 3.35 (dd, *J* = 14.1, 8.2 Hz, 1H), 2.39 (s, 3H), 1.85 (q, *J* = 7.6 Hz, 1H), 1.59 (dd, *J* = 14.1, 10.8 Hz, 1H), 0.91 (d, *J* = 7.6 Hz, 3H). ^13^C-NMR (151 MHz, CDCl_3_): *δ* 186.30, 172.36, 163.78, 150.34, 146.93, 146.61, 143.85, 142.57, 142.48, 142.16, 137.45, 135.48, 127.82, 126.01, 85.89, 80.06, 79.52, 69.76, 48.85, 44.77, 42.05, 40.04, 28.95, 24.11, 14.86. HR-MS(ESI) *m/z*: 454.1259, [M + Na]^+^ (Calcd for C_25_H_21_NNaO_6_: 454.1261).

*(3R,4aR,9S,10aS,11S)-5,11-dimethyl-1,7-dioxo-2a,2a1,3,4a,7,9,10,10a1-octahydro-1H-2,4-dioxa-3,10a-methanocyclohepta[bc]cyclopenta[jk]acenaphthylen-9-yl 3,5,6-trimethylpyrazine-2-carboxylate (**11f**).* Yield: 76% (10.8 mg).

^1^H-NMR (600 MHz, CDCl_3_): *δ* 7.21 (d, *J* = 2.2 Hz, 1H), 6.99 (s, 1H), 6.21 (ddd, *J* = 10.5, 8.1, 2.0 Hz, 1H), 5.38 (d, *J* = 5.4 Hz, 1H), 5.22 (t, *J* = 5.8 Hz, 1H), 4.01 (d, *J* = 5.6 Hz, 1H), 3.60 (d, *J* = 8.7 Hz, 1H), 3.42 (dt, *J* = 8.7, 5.7 Hz, 1H), 3.36 (dd, *J* = 14.0, 8.1 Hz, 1H), 2.79 (s, 3H), 2.60 (d, *J* = 3.3 Hz, 6H), 2.38 (s, 3H), 1.86 (q, *J* = 7.6 Hz, 1H), 1.50 (dd, *J* = 14.0, 10.8 Hz, 1H), 0.91 (d, *J* = 7.6 Hz, 3H). ^13^C-NMR (151 MHz, CDCl_3_): *δ* 186.41, 172.43, 164.37, 155.50, 152.28, 149.89, 146.52, 143.74, 142.63, 142.59, 142.16, 138.21, 135.74, 85.90, 80.07, 79.51, 69.56, 48.81, 44.82, 42.05, 40.04, 28.97, 24.07, 22.81, 22.41, 21.86, 14.90. HR-MS(ESI) *m/z*: 497.1679, [M + Na]+ (Calcd for C_27_H_26_N_2_NaO_6_: 497.1683).

To a stirred solution of **12** (10 mg, 0.03 mmol) in CH_2_Cl_2_, DMP (17 mg, 0.04 mmol) was added. The reaction mixture was stirred at room temperature for 5 h and then diluted with CH_2_Cl_2_. The organic phase was dried (Na_2_SO_4_) and filtered. Concentration of the filtrate gave a residue that was fractionated by column chromatography hexanes-EtOAc (1:1) to afford **13** (7.6 mg, 78%) as a yellow solid.

*(3R,4aR,10aS,11S)-5,11-dimethyl-2a,3,4a,10a1-tetrahydro-1H-2,4-dioxa-3,10a-methanocyclohepta[bc]cyclopenta[jk]acenaphthylene-1,7,9(2a1H,10H)-trione (**13**).*

^1^H-NMR (600MHz, CDCl_3_): *δ* 7.45 (d, *J* = 2.7 Hz, 1H), 6.92 (s, 1H), 5.39 (d, *J* = 5.1 Hz, 1H), 5.27 (t, *J* = 5.6 Hz, 1H), 4.07 (d, *J* = 5.6 Hz, 1H), 3.52 (d, *J* = 8.8 Hz, 1H), 3.45 (dt, *J* = 8.8, 5.3 Hz, 1H), 3.32 (d, *J* = 20.2 Hz, 1H), 2.71 (d, *J* = 20.2 Hz, 1H), 2.39 (s, 3H), 2.13 (q, *J* = 7.6 Hz, 1H), 0.97 (d, *J* = 7.6 Hz, 3H). ^13^C-NMR (151 MHz, CDCl_3_): *δ* 195.24, 187.10, 171.84, 147.25, 144.85, 142.44, 141.23, 139.49, 136.09, 85.31, 79.80, 79.44, 50.49, 45.15, 40.28, 38.90, 38.71, 23.90, 15.31. HR-MS(ESI) *m/z*: 347.0886, [M + H]^+^ (Calcd for C_19_H_16_NaO_5_: 347.0890).

### 4.2. Cell Culture

HCT-116 (Human colon cancer cell line), A375 (human melanoma cancer cell line), A-549 (human lung adenocarcinoma cell line), Huh-7 (human hepatoma cancer cell line), and L-02 (normal hepatic cell line) were purchased from the Cell Bank of Shanghai Institute of Biochemistry and Cell Biology, Chinese Academy of Sciences (Shanghai, China). The cells were cultured in Dulbecco’s modified Eagle medium (DMEM; NY, USA), which were all supplemented with 10% fetal bovine serum (FBS; NY, USA) at 37 °C with 5% CO_2_.

### 4.3. MTT Assay

The cytotoxic activity was evaluated using the MTT assay. Briefly, cells were seeded in 96-well plates at a density of 5 × 10^3^ cells/well in 100 μL of DMEM medium at 37 °C for 24 h. Stock solutions of test compounds were prepared in DMSO at concentration of 50 mM, and twofold serial dilution with a culture medium was performed to obtain appropriate concentrations right before the assay. After that, the cells were incubated with synthetic derivatives or cisplatin as a positive control at the various concentrations (0.78125-50 μM) for 48 h. Then, 20 μL of 3-(4,5-Dimethylthiazol-2-yl)-2,5-diphenyltetrazolium bromide (MTT, Aladdin, Shanghai, China) solution (5 mg/mL in PBS) was added, and then the cells were incubated at 37 °C for an additional 4 h. The supernatant was removed, and 150 μL of dimethyl sulfoxide was added to dissolve the MTT formazan. The optical density (OD) was measured on a microplate reader (SpectraMax Plus 384, Molecular Devices, Sunnyvale, CA, USA) at a wavelength of 570 nm. The cytotoxic activity was expressed as the IC_50_ values. The above tests were repeated three times in parallel. Vehicle DMSO (0.1%) was used as a negative control.

## Figures and Tables

**Table 1:** The in-vitro cytotoxicity of the harringtonolide (HO) analogues.

Compounds	IC_50_ (μM)			
	HCT-116	A375	A549	Huh-7
**HO (1)**	0.61 ± 0.03	1.34 ± 0.23	1.67 ± 0.23	1.25 ± 0.08
**2**	>50	>50	>50	>50
**3**	>50	>50	>50	>50
**4**	>50	>50	>50	>50
**5**	>50	>50	>50	>50
**6**	0.86 ± 0.05	4.85 ± 0.42	5.47 ± 0.13	1.19 ± 0.08
**7**	>50	>50	>50	>50
**8**	2.19 ± 0.23	3.40 ± 0.22	43.40 ± 1.08	30.75 ± 0.60
**9**	>50	>50	>50	>50
**10**	2.29 ± 0.27	5.79 ± 1.26	8.25 ± 0.20	6.34 ± 0.32
**11a**	5.95 ± 0.45	30.13 ± 1.58	27.49 ± 0.89	>50
**11b**	>50	>50	>50	>50
**11c**	10.98 ± 0.34	16.42 ± 1.92	23.25 ± 0.67	>50
**11d**	>50	>50	>50	>50
**11e**	5.47 ± 0.27	7.08 ± 0.42	17.98 ± 4.18	>50
**11f**	10.77 ± 0.42	18.56 ± 2.23	25.95 ± 0.68	>50
**12**	31.88 ± 0.69	40.42 ± 7.58	34.72 ± 1.94	>50
**13**	>50	>50	>50	>50
Cisplatin	4.48 ± 0.18	4.04 ± 0.23	8.53 ± 0.98	15.04 ± 0.15

**Table 2:** Cytotoxicity of compounds **1**, **6** and **10** on L-02 cells.

Compound	IC_50_ (μM)		SI *^a^*
	Huh-7	L-02	
**6**	1.19 ± 0.08	67.2 ± 0.43	56.5
**10**	6.34 ± 0.32	22.33 ± 1.11	3.5
**HO (1)**	1.25 ± 0.08	3.46 ± 0.36	2.8

*^a^* Selectivity index is defined as IC_50_ (L-02)/IC_50_ (Huh-7).

## Data Availability

The data presented in this study are available on request from the corresponding author.

## Supplementary Materials

The following are available online. Scheme S1: Possible mechanism for the formation of the derivatives **7**; Scheme S2: Possible mechanism for the formation of the derivatives **9**; Figure S1: NMR spectra for all compounds; Figure S2: Purity assay for all compounds [29,30,31,32].

## Sample Availability

Samples of the compounds are not available from the authors.

## References

[1] He Y.R., Shen Y.H., Shan L., Yang X., Wen B., Ye J., Yuan X., Li H.L., Xu X.K., Zhang W.D. (2015). Diterpenoid Lanceolatins A–G from *Cephalotaxus Lanceolata* and Their Anti-Inflammatory and Anti-Tumor Activities. RSC Adv..

[2] Huang Q., Guo K., Liu Y., Liu Y., Li W., Geng H., Wang Y., Li S. (2020). Diterpenoids and Flavonoids from the Twigs of *Cephalotaxus Fortunei Var. Alpina*. Chem. Biodivers.

[3] Zhao J.X., Fan Y.Y., Xu J.B., Gan L.S., Xu C.H., Ding J., Yue J.M. (2017). Diterpenoids and Lignans from *Cephalotaxus Fortunei*. J. Nat. Prod..

[4] Zhao C.X., Li B.Q., Shao Z.X., Li D.H., Jing Y.K., Li Z.L., Hua H.M. (2019). Cephasinenoside A, a New Cephalotane Diterpenoid Glucoside from *Cephalotaxus Sinensis*. Tetrahedron Lett..

[5] Li Y., Wang Y., Shao Z., Zhao C., Jing Q., Li D., Lin B., Jing Y., Li Z., Hua H. (2020). Diterpenoids from *Cephalotaxus Fortunei Var. Alpina* and Their Cytotoxic Activity. Bioorg.c Chem..

[6] Buta J.G., Flippen J.L., Lusby W.R. (1978). Harringtonolide, a Plant Growth Inhibitory Tropone from *Cephalotaxus Harringtonia* (Forbes) K. Koch. J. Org. Chem..

[7] Kang S.Q., Cai S.Y., Teng L. (1981). The Antiviral Effect of Hainanolide, A Compound Isolated from *Cephaloyaxis Hainanensis*. Acta Pharm Sin..

[8] Ni L., Zhong X.H., Chen X.J., Zhang B.J., Bao M.F., Cai X.H. (2018). Bioactive Norditerpenoids from *Cephalotaxus Fortunei Var. Alpina* and *C. Lanceolata*. Phytochemistry.

[9] Evanno L., Jossang A., Pouplin J.N., Delaroche D., Herson P., Seuleiman M., Bodo B., Nay B. (2008). Further Studies of the Norditerpene (+)-Harringtonolide Isolated from *Cephalotaxus Harringtonia Var. Drupacea*: Absolute Configuration, Cytotoxic and Antifungal Activities. Planta. Med..

[10] Zhang H.J., Hu L., Ma Z., Li R., Zhang Z., Tao C., Cheng B., Li Y., Wang H., Zhai H. (2016). Total Synthesis of the Diterpenoid (+)-Harringtonolide. Angew. Chem..

[11] Ma Z., Cheng B., Zhai H. (2014). Synthetic Studies Toward Harringtonolide. Asian J. Org. Chem..

[12] Abdelkafi H., Herson P., Nay B. (2012). Asymmetric Synthesis of the Oxygenated Polycyclic System of (+)-Harringtonolide. Org. Lett..

[13] O’Sullivan T.P., Zhang H., Mander L.N. (2007). Model Studies toward the Synthesis of the Bioactive Diterpenoid, Harringtonolide. Org. Biomol. Chem..

[14] Frey B., Wells A.P., Rogers D.H., Mander L.N. (1998). Synthesis of the Unusual Diterpenoid Tropones Hainanolidol and Harringtonolide. J. Am. Chem. Soc..

[15] Zhang M., Liu N., Tang W. (2013). Stereoselective Total Synthesis of Hainanolidol and Harringtonolide via Oxidopyrylium-Based [5 + 2] Cycloaddition. J. Am. Chem. Soc..

[16] Zhang H.B., Appels D.C., Hockless D.C.R., Mander L.N. (1998). A New Approach to the Total Synthesis of the Unusual Diterpenoid Tropone, Harringtonolide. Tetrahedron Lett..

[17] Abdelkafi H., Evanno L., Herson P., Nay B. (2011). Synthetic Studies toward the Cytotoxic Norditerpene (+)-Harringtonolide: Setting up Key-Stereogenic Centers of the Cyclohexane Ring D. Tetrahedron Lett..

[18] Frey B., Mander L.N., Hockless D.C.R. (1998). Ether Formation by Intramolecular Attack of Hydroxy on Cyclopropyl Rings: A Model for the Formation of the Tetrahydrofuran Moiety in the Diterpenoid, Harringtonolide. J. Chem. Soc. Perkin Trans. 1.

[19] Sun N.J., Xue Z., Liang X.T., Huang L. (1979). Studies on the Structure of A New Antitumor Agent-Hainanolide. Acta Pharm. Sin..

[20] Fan Y.Y., Xu J.B., Liu H.C., Gan L.S., Ding J., Yue J.M. (2017). Cephanolides A–J, Cephalotane-Type Diterpenoids from *Cephalotaxus Sinensis*. J. Nat. Prod..

[21] Ge Z.P., Liu H.C., Wang G.C., Liu Q.F., Xu C.F., Xu C.F., Ding J., Fan Y.Y., Yue J.M. (2019). 17-*nor*-Cephalotane-Type Diterpenoids from *Cephalotaxus Fortunei*. J. Nat. Prod..

[22] Du J., Chiu M.H., Nie R.L. (1999). Two New Lactones from *Cephalotaxus Fortunei Var. Alpnia*. J. Nat. Prod..

[23] Ni G., Zhang H., Fan Y.Y., Liu H.C., Ding J., Yue J.M. (2016). Mannolides A–C with an Intact Diterpenoid Skeleton Providing Insights on the Biosynthesis of Antitumor Cephalotaxus Troponoids. Org. Lett..

[24] Wu G.R., Xu B., Yang Y.Q., Zhang X.Y., Fang K., Ma T., Wang H., Xue N.N., Chen M., Guo W.B. (2018). Synthesis and Bio-logical Evaluation of Podophyllotoxin Derivatives as Selective Antitumor Agents. Eur. J. Med. Chem..

[25] Stern P.H., Hoffman R.M. (1986). Enhanced In Vitro Selective Toxicity of Chemotherapeutic Agents for Human Cancer Cells Based on a Metabolic Defect. J. Natl. Cancer Inst..

[26] Haglund C., Åleskog A., Nygren P., Gullbo J., Höglund M., Wickström M., Larsson R., Lindhagen E. (2012). In Vitro Evaluation of Clinical Activity and Toxicity of Anticancer Drugs Using Tumor Cells from Patients and Cells Representing Normal Tissues. Cancer Chemother. Pharmacol..

[27] You Y.J., Kim Y., Nam N.H., Ahn B.Z. (2003). Synthesis and Cytotoxic Activity of A-Ring Modified Betulinic Acid Derivatives. Bioorg. Med. Chem. Lett..

[28] Rao Z., Liu X., Zhou W., Yi J., Li S.S. (2011). Synthesis and Antitumour Activity of β-Hydroxyisovalerylshikonin Analogues. Eur. J. Med. Chem..

[29] Demidov M.R., Lapshina M.Y., Osipov D.V., Osyanin V.A., Klimochkin Y.N. (2017). Oxidative Rearrangement of 4*H*-Chromenes to 2-Aroylbenzofurans in the Presence of Selenium Dioxide. Chem. Heterocycl. Comp..

[30] Morita S., Yoshimura T., Matsuo J. (2019). Intramolecular Büchner Reaction and Oxidative Aromatization with SeO2 or O2. Chem. Pharm. Bull..

[31] Karimi S., Ma S., Ramig K., Greer E.M., Szalda D.J., Subramaniam G. (2015). Oxidative Ring-Contraction of 3*H*-1-Benzazepines to Quinoline Derivatives. Tetrahedron Lett..

[32] Maier W.F., Roth W., Thies I., Schleyer P.V.R. (1982). Hydrogenolysis, iv. gas phase decarboxylation of carboxylic acids. Chem. Ber..

