# Supplementary material for: Semi-Synthesis of Harringtonolide Derivatives and Their Antiproliferative Activity
Source: Molecules. 2021 Mar 4;26(5):1380. doi: 10.3390/molecules26051380 (PMC7961763; doi:10.3390/molecules26051380)
Supplement: Supplementary file 1 [file molecules-26-01380-s001.pdf]

Supplementary Materials

Semi-synthesis of Harringtonolide Derivatives and Their Antiproliferative Activity

Xiutao Wu [1], Lijie Gong [1], Chen Chen [1], Ye Tao [1], Wuxi Zhou [1], Lingyi Kong [1,*] and Jianguang Luo [1,*]

[1] State Key Laboratory of Natural Medicines, Jiangsu Key Laboratory of Bioactive Natural Product Research, School of Traditional Chinese Pharmacy, China Pharmaceutical University, 24 Tong Jia Xiang, Nanjing, 210009, PR China; wxtcpuer@163.com (X.W.); jieligong313@163.com (L.G.); chenchen02544@163.com (C.C.); tycpuer@163.com (T.Y.); zhouwuxi1988@163.com (W.Z.);

* Correspondence: cpu_lykong@126.com (L.K.); luojg@cpu.edu.cn (J.L.); Tel.: +86-025-8327-1405 (L.K. & J.L.)

Table of Contents

Scheme S1 2
Scheme S2 2
Figure S1： NMR spectra for all compounds 3
Compound 2 3
Compound 3 4
Compound 4 5
Compound 5 6
Compound 6 7
Compound 7 8
Compound 8 9
Compound 9 10
Compound 10 11
ROESY Spectra of compound 10 12
Compound 10a 13
Compound 10b 14
Compound 10c 15
Compound 10d 16
Compound 10e 17
Compound 10f 18
Compound 11 19
Compound 12 20
References 21

Scheme S1

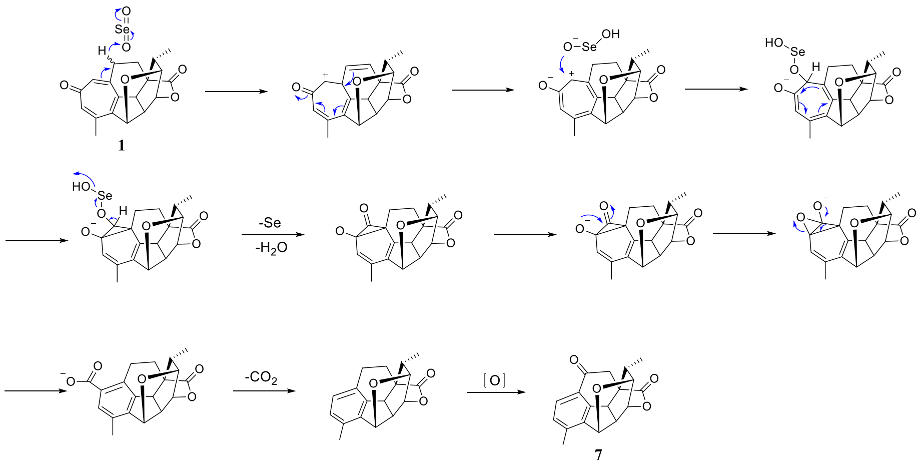

A possible mechanism for the formation of **7** is presented in Scheme S1. The initial oxidation of allylic in HO (**1**) by SeO_2 was accompanied by the formation of $HSeO_2^-$ anion [1] and carbocation, which would rearrange to cycloheptatriene carbocation. Subsequently, $HSeO_2^-$ attacked the cycloheptatriene carbocation, while that followed by electrocyclic annulation to give cyclopropanes intermediate. The subsequent elimination of Se and H_2O led to the cyclopropenone intermediate [2-3], which then attacked by oxygen anion. Subsequent rearrangement occurs in the manner as indicated arrows, giving carboxylic acid intermediate. Further decarboxylation [4] of this intermediate and oxidation by SeO_2 would yield compound **7**.

Scheme S2

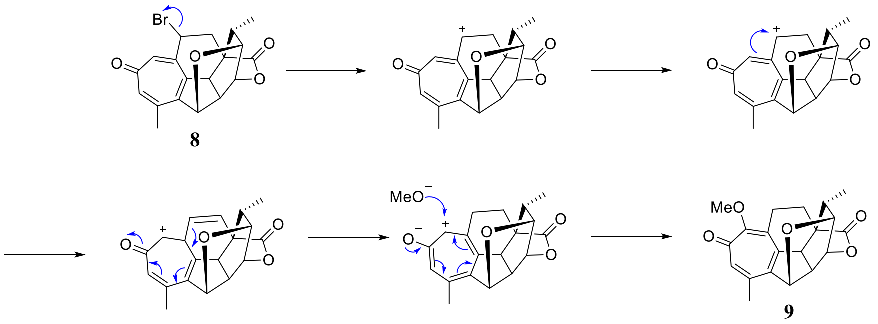

A possible mechanism for the formation of **9** is presented in Scheme S2. Under the attacked of nucleophile (methoxy anion), leaving of bromine atoms in compound **8** yield an allylic carbocation, which then rearranged to a more stable form–cycloheptatriene carbocation too. Subsequently, this carbocation intermediate was attacked by methoxy anion, and then further electron transfer occurs in the manner as indicated arrows to produce **9**.

Figure S1：NMR spectra for all compounds

Compound 2

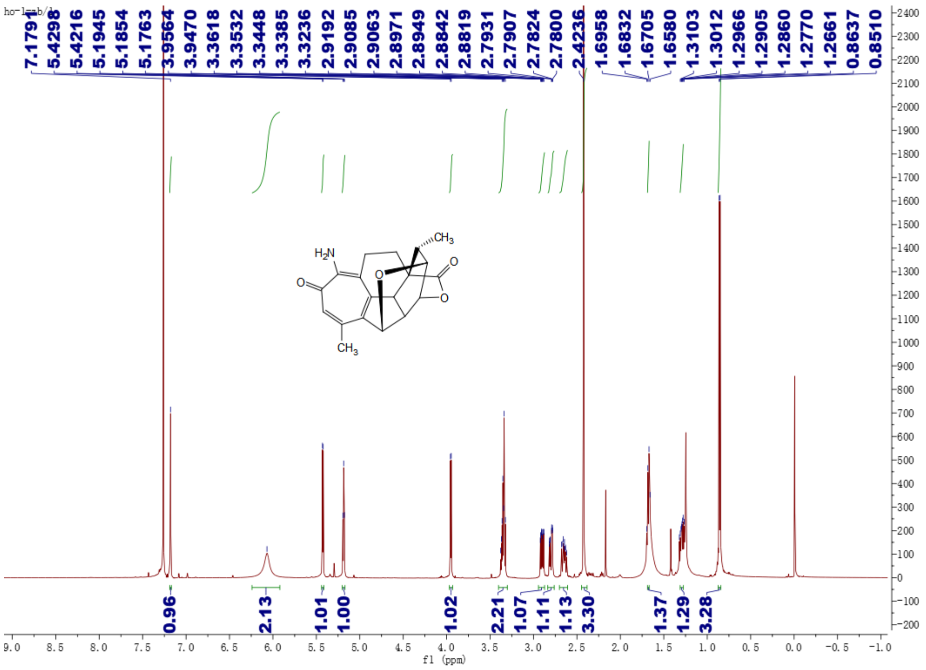

^{1}H-NMR spectrum (600 MHz, $CDCl_3$) for 2

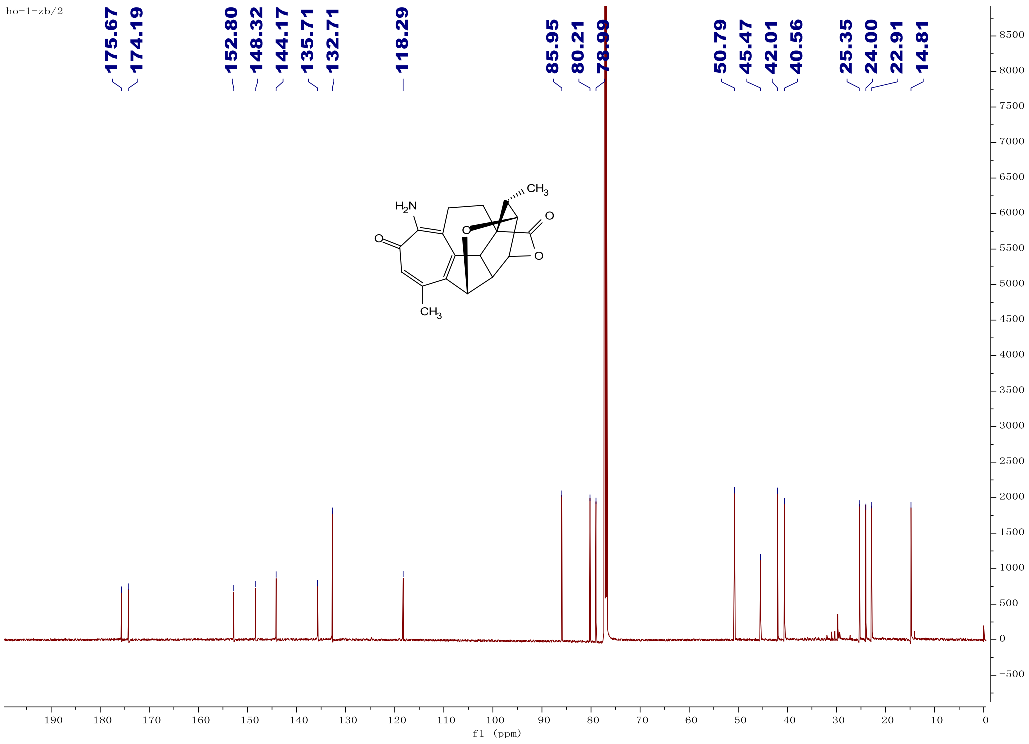

^{13}C-NMR spectrum (151 MHz, $CDCl_3$) for 2

Compound 3

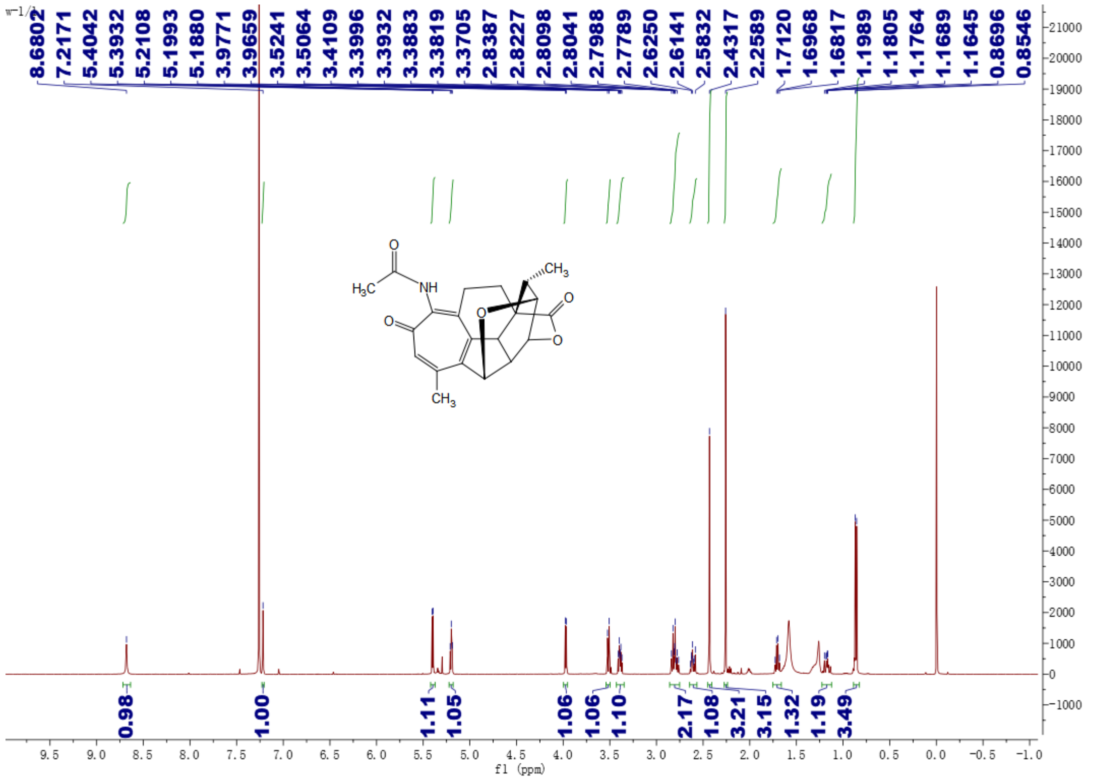

^{1}H-NMR spectrum (500 MHz, $CDCl_3$) for 3

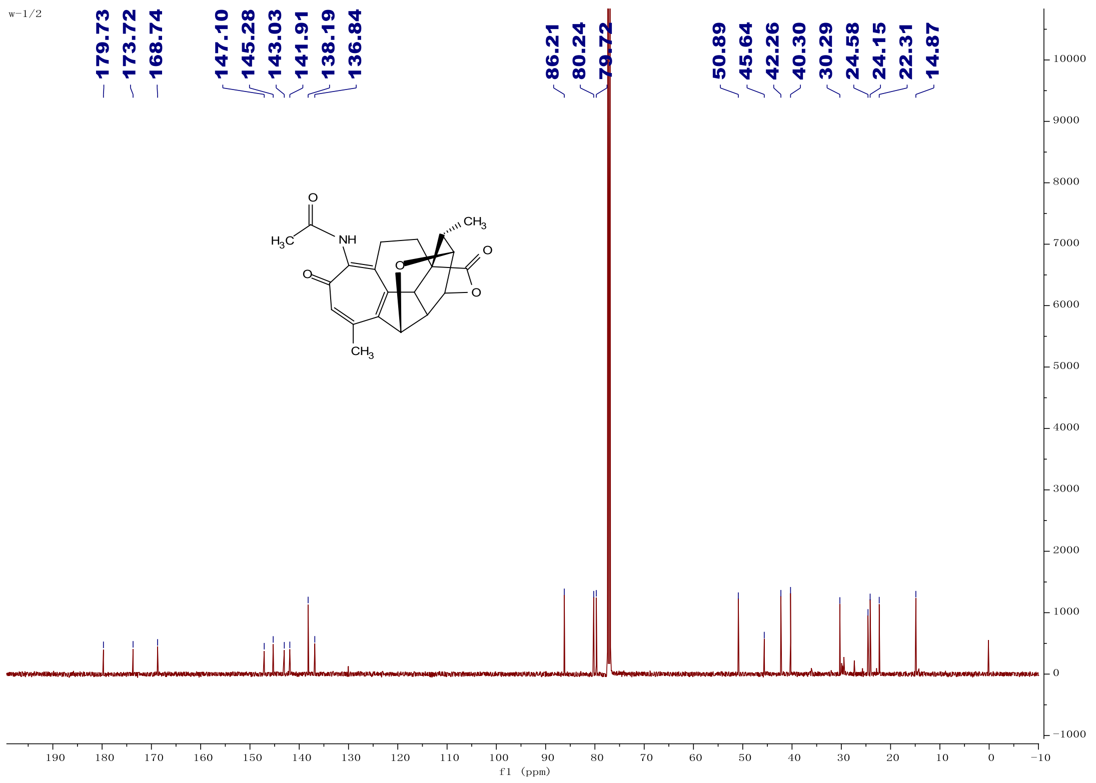

^{13}C-NMR spectrum (126 MHz, $CDCl_3$) for 3

Compound 4

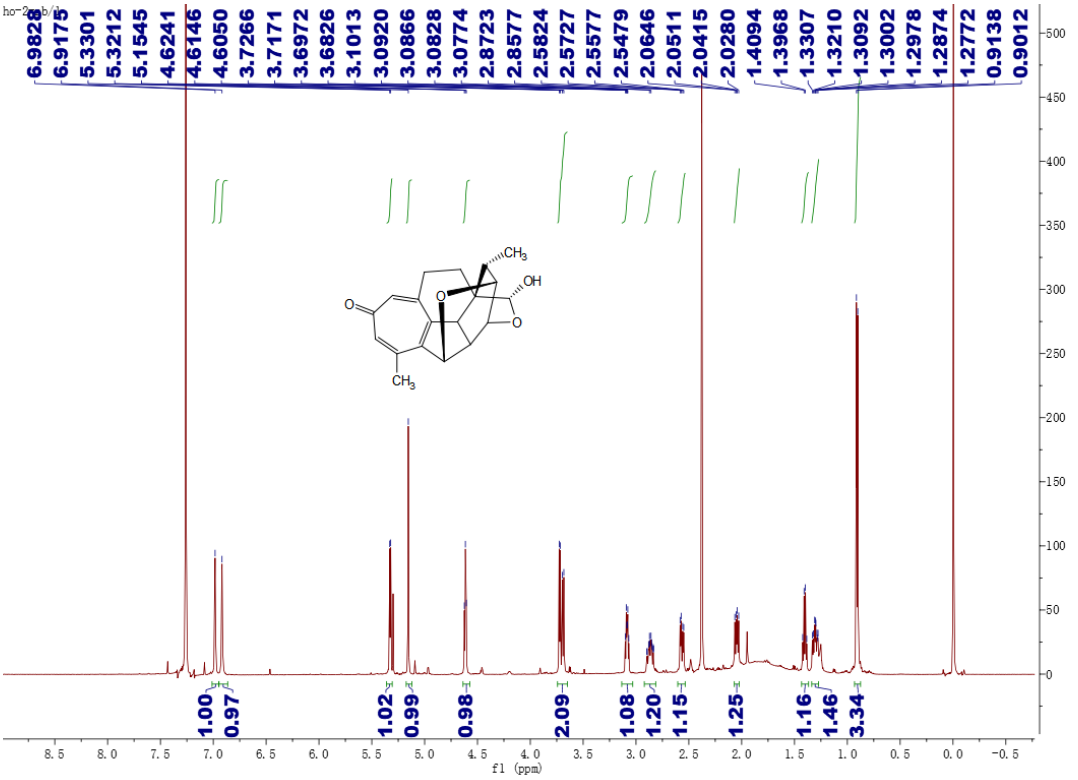

^{1}H-NMR spectrum (600 MHz, $CDCl_3$) for 4

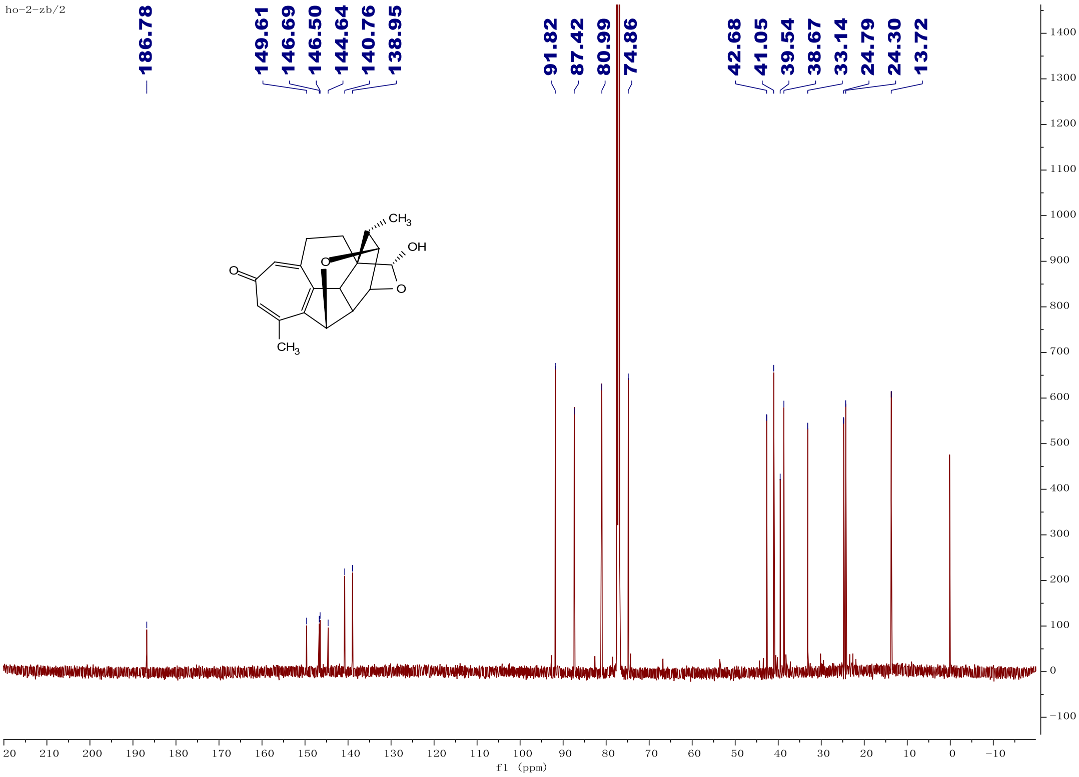

^{13}C-NMR spectrum (151 MHz, $CDCl_3$) for 4

Compound 5

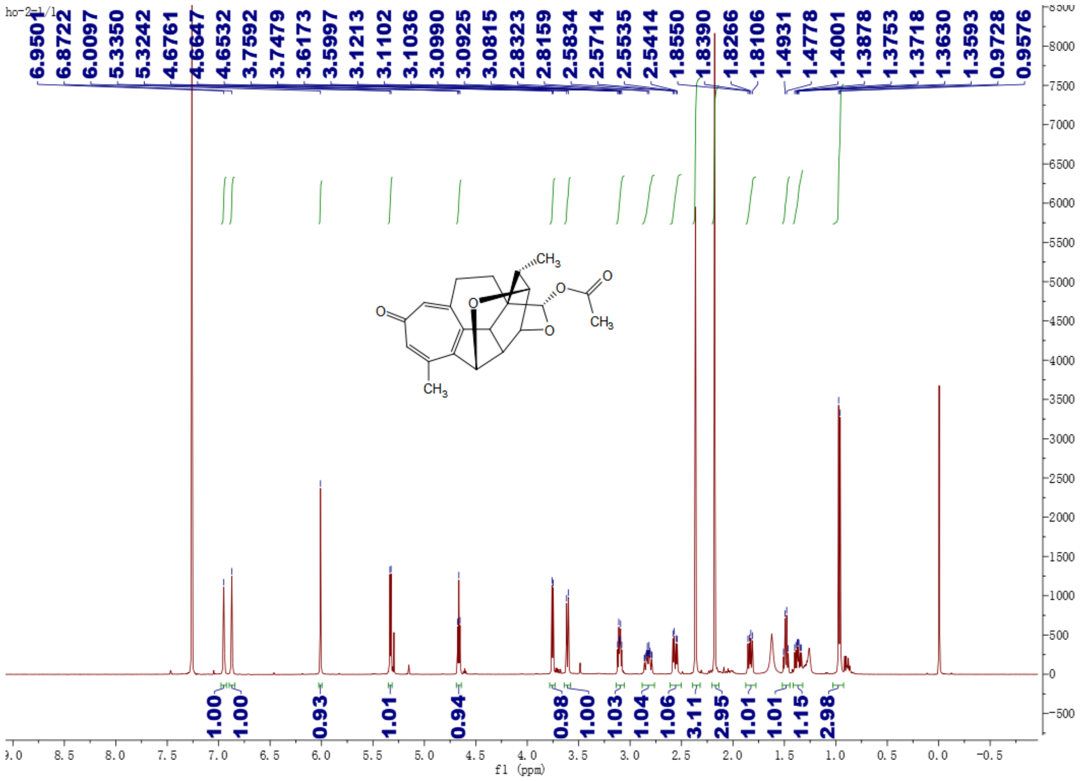

^{1}H-NMR spectrum (500 MHz, $CDCl_3$) for 5

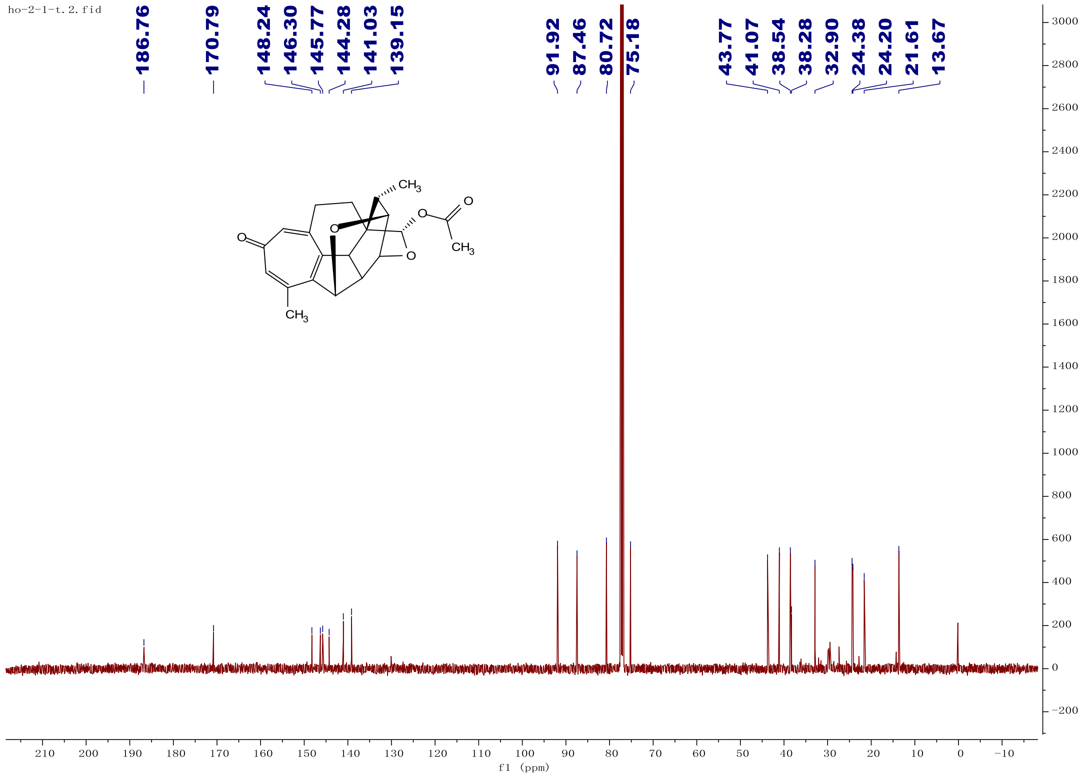

^{13}C-NMR spectrum (151 MHz, $CDCl_3$) for 5

Compound 6

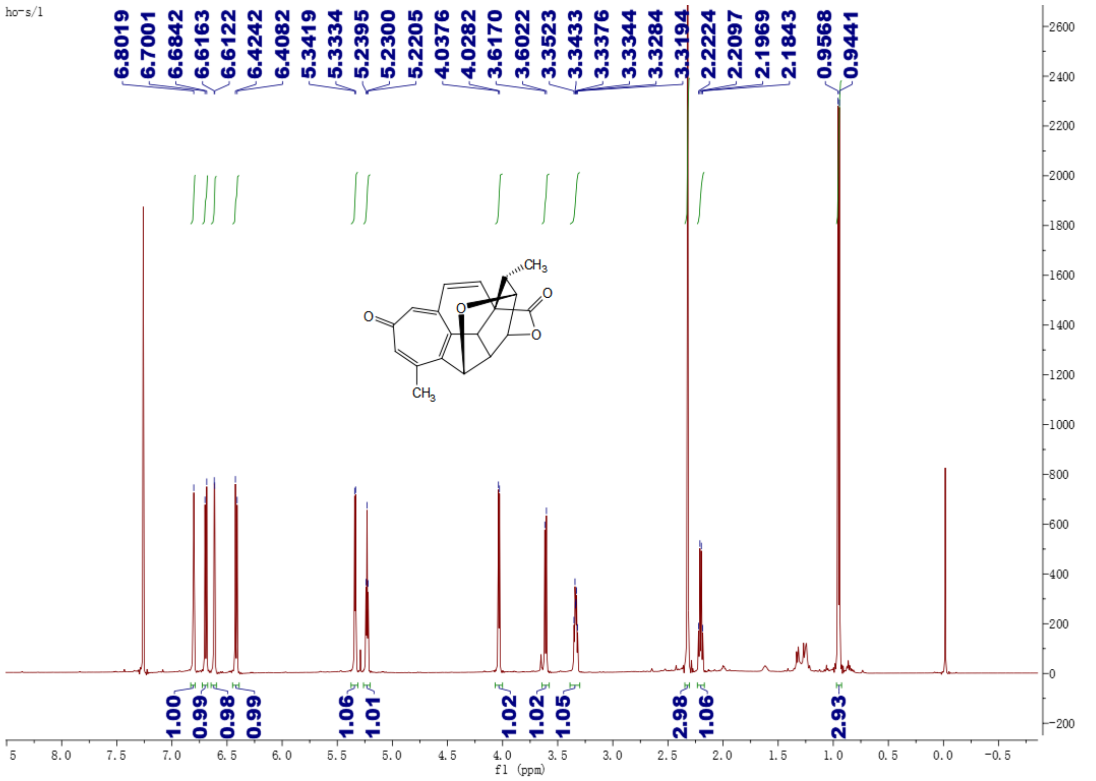

^{1}H-NMR spectrum (600 MHz, CDCl$_3$) for 6

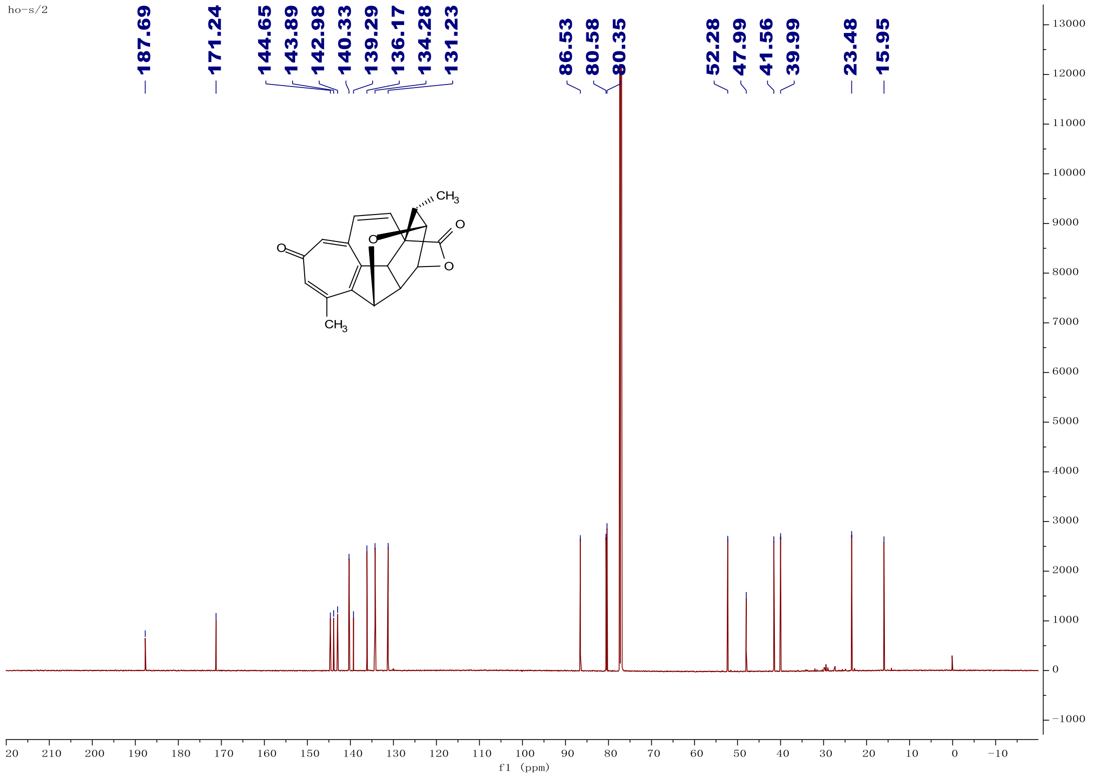

^{13}C-NMR spectrum (151 MHz, CDCl$_3$) for 6

Compound 7

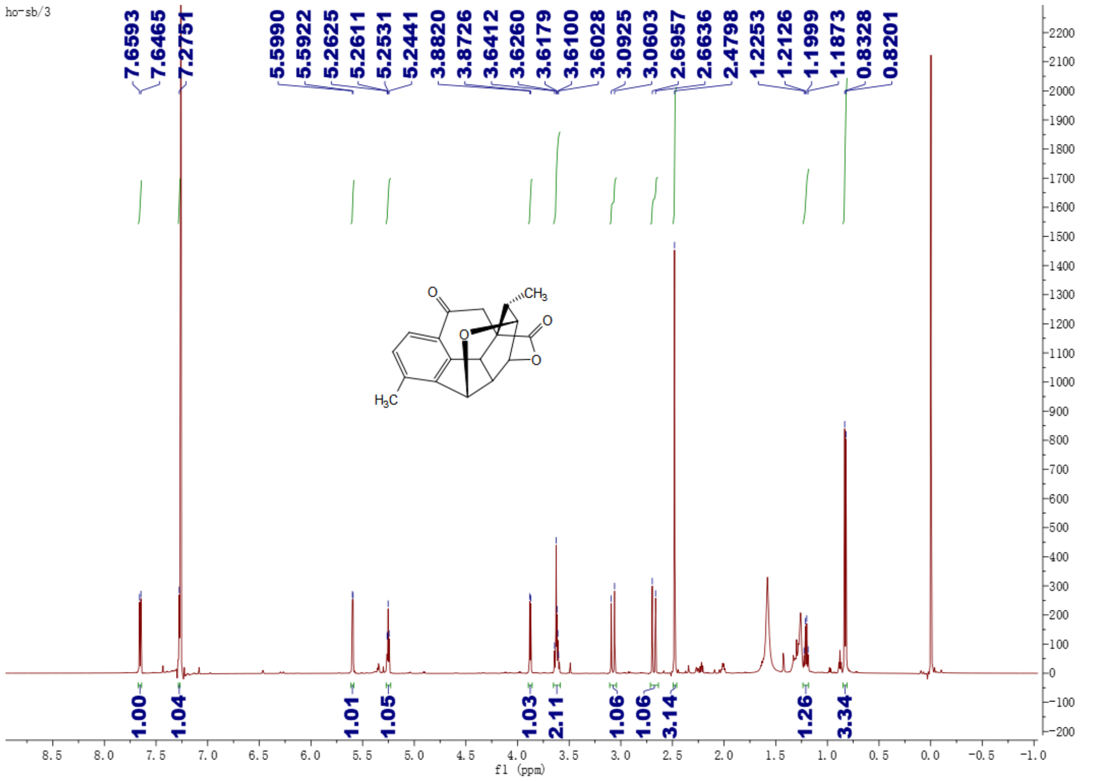

^{1}H-NMR spectrum (600 MHz, $CDCl_3$) for 7

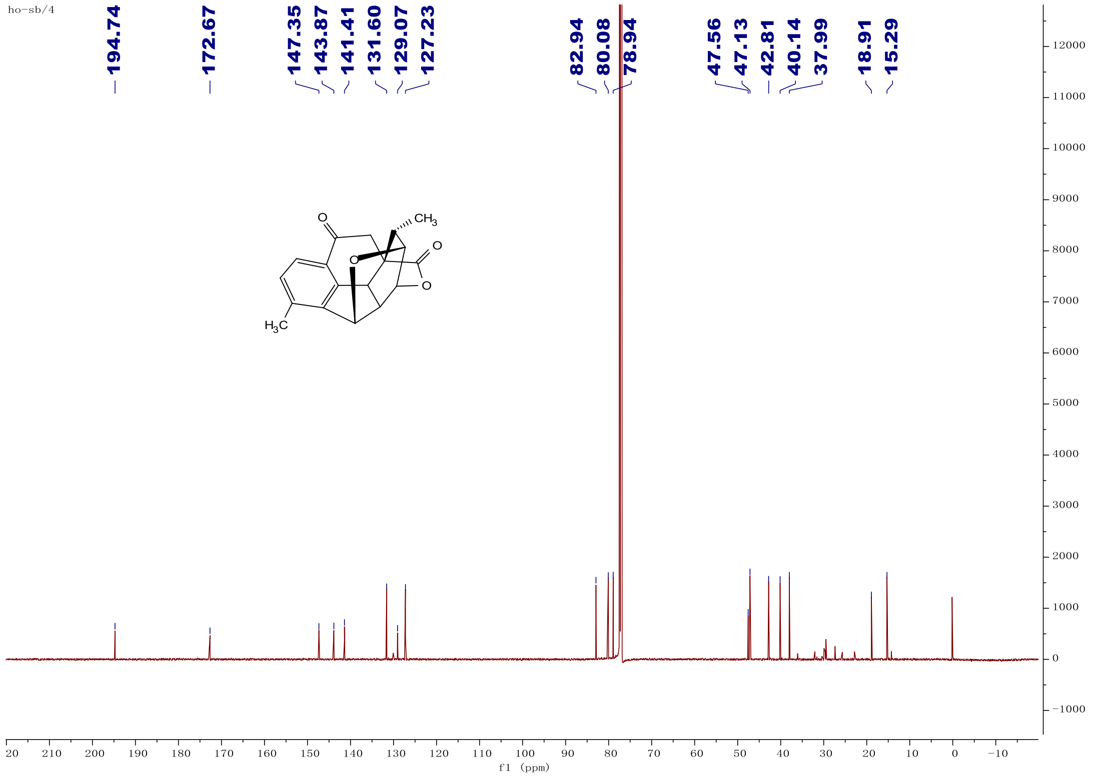

^{13}C-NMR spectrum (151 MHz, $CDCl_3$) for 7

Compound 8

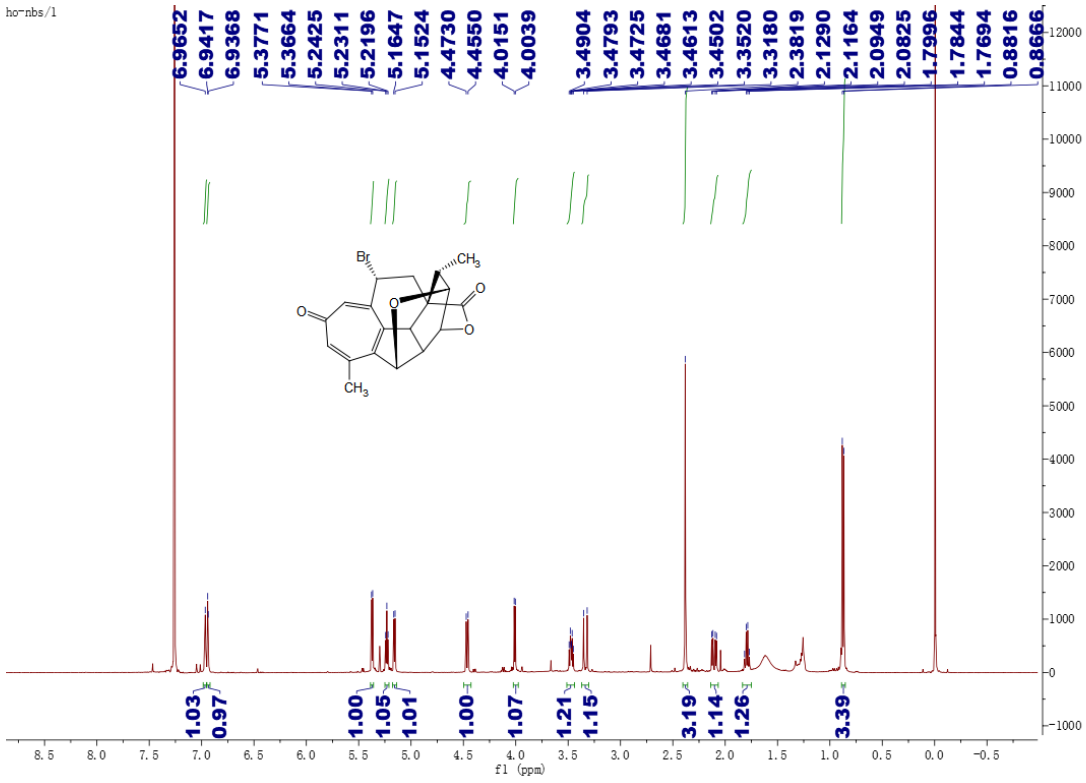

^{1}H-NMR spectrum (500 MHz, $CDCl_3$) for 8

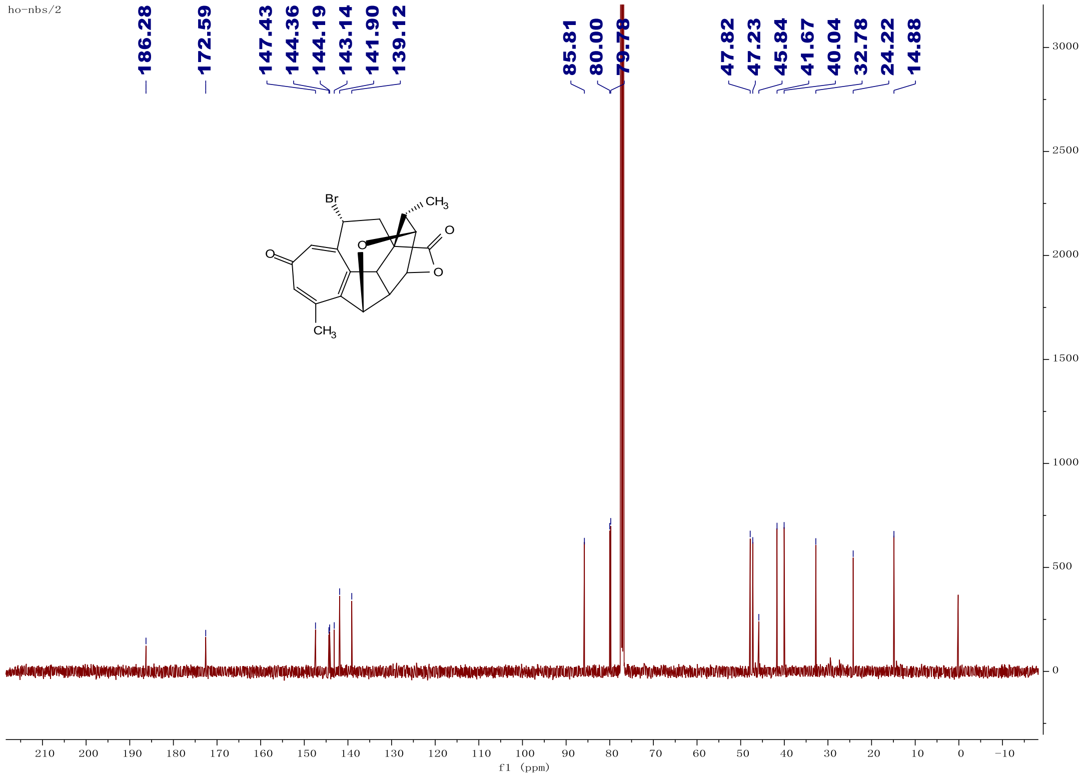

^{13}C-NMR spectrum (126 MHz, $CDCl_3$) for 8

Compound 9

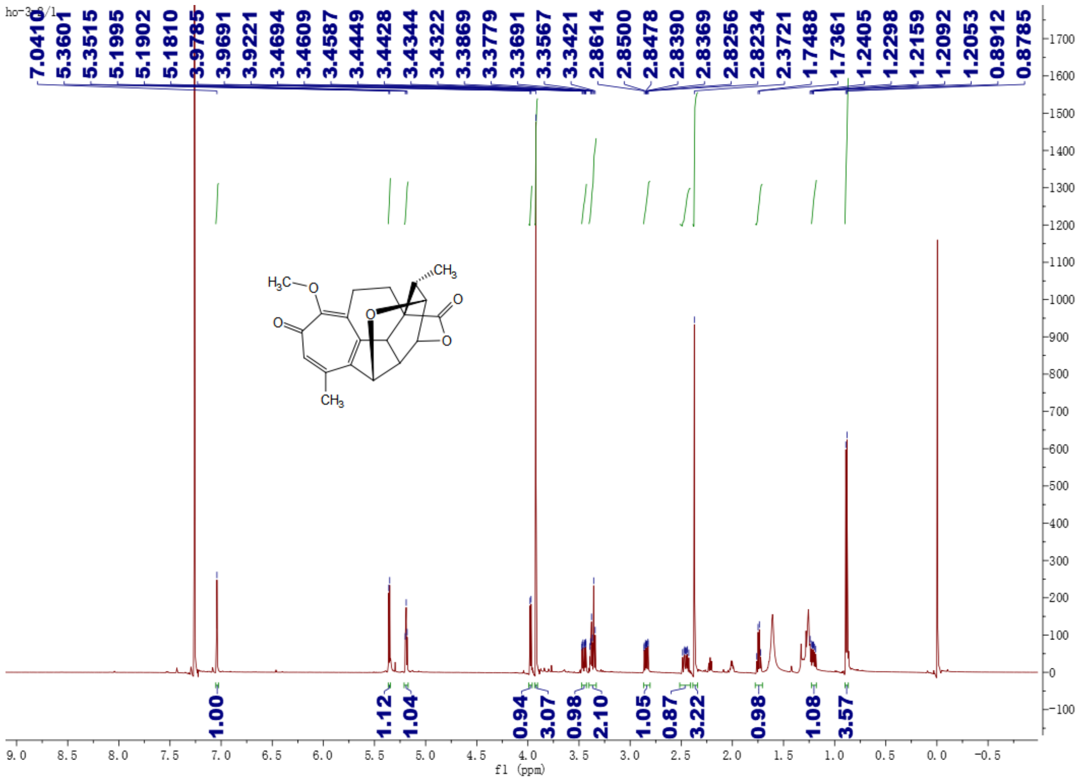

^{1}H-NMR spectrum (600 MHz, $CDCl_3$) for 9

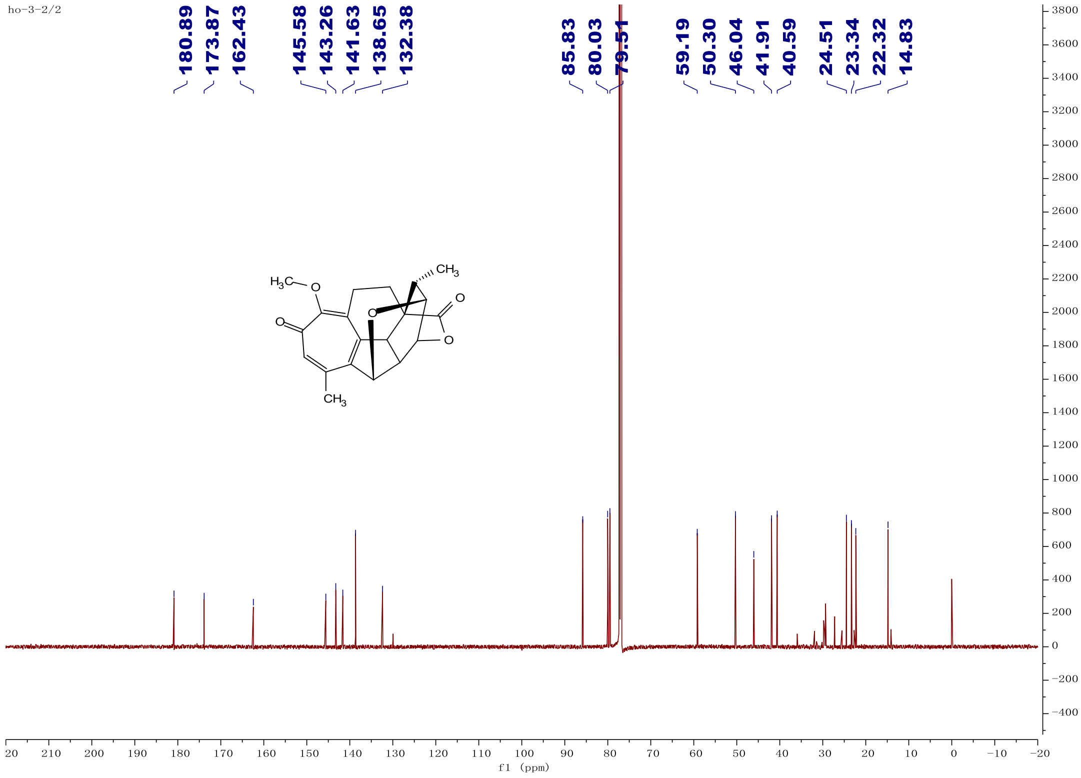

^{13}C-NMR spectrum (151 MHz, $CDCl_3$) for 9

Compound 10

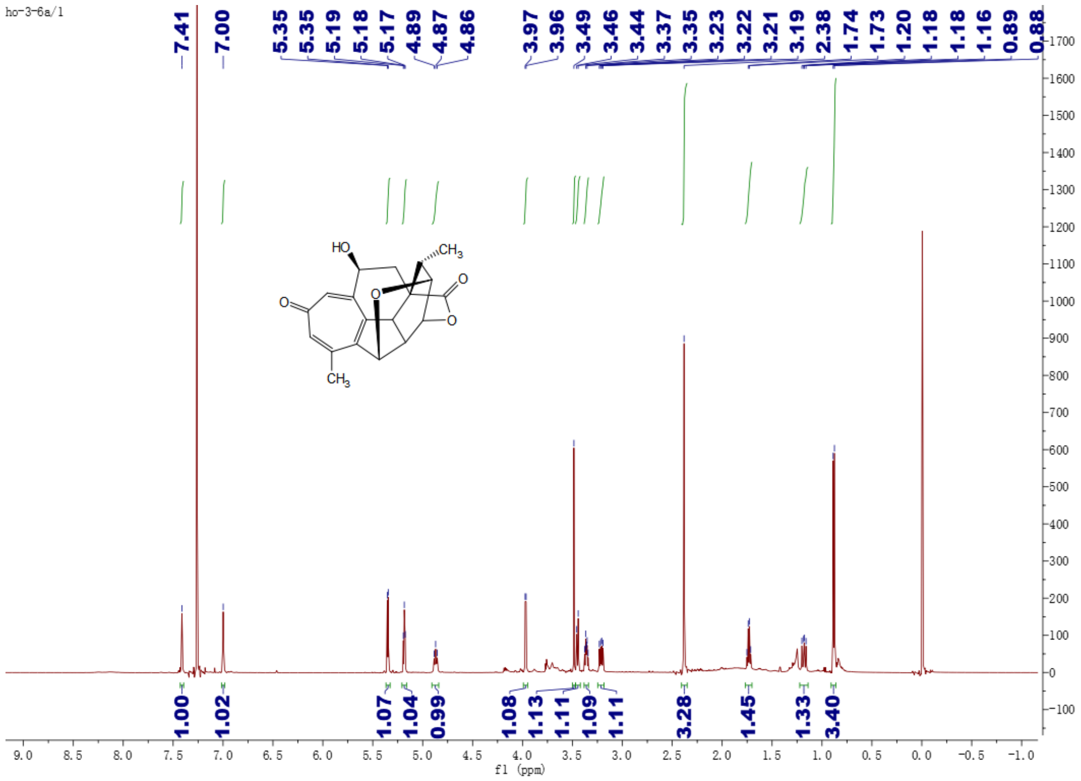

^{1}H-NMR spectrum (600 MHz, $CDCl_3$) for 10

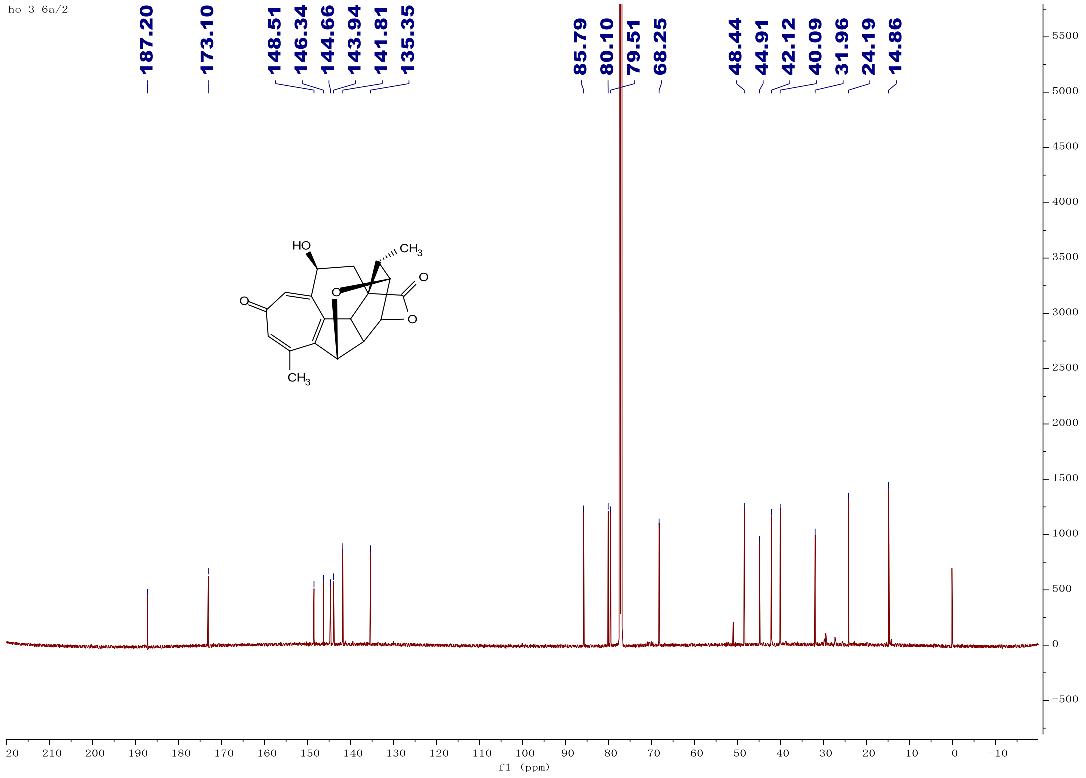

^{13}C-NMR spectrum (151 MHz, $CDCl_3$) for 10

ROESY Spectra of compound 10

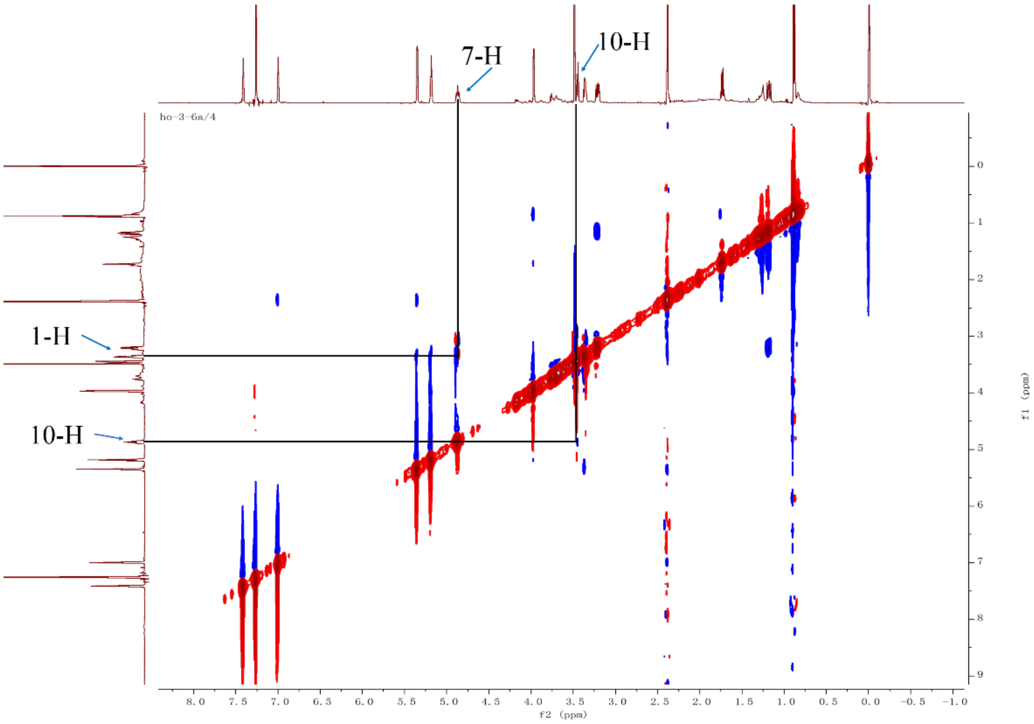

ROESY Spectra of compound 10

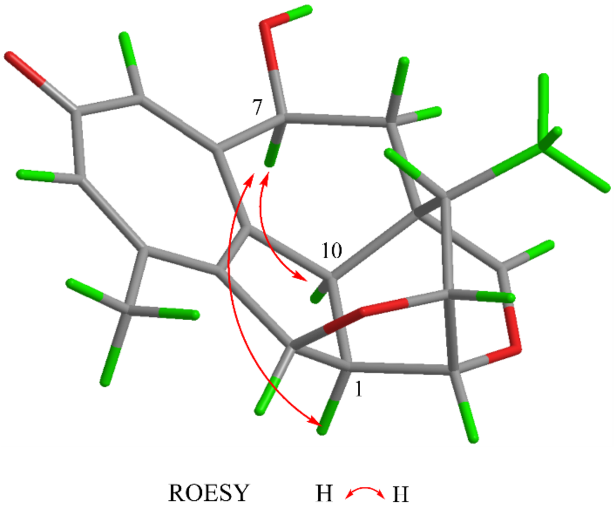

ROESY correlations of compound 10

Compound 10a

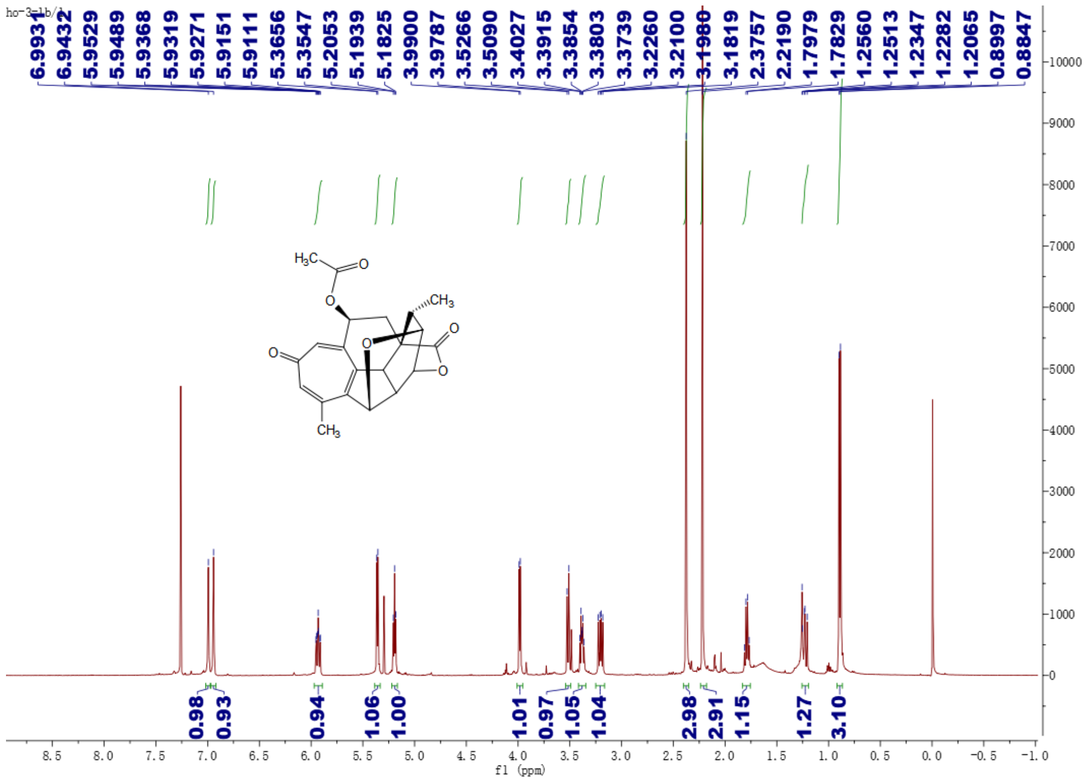

^{1}H-NMR spectrum (500 MHz, CDCl3) for 10a

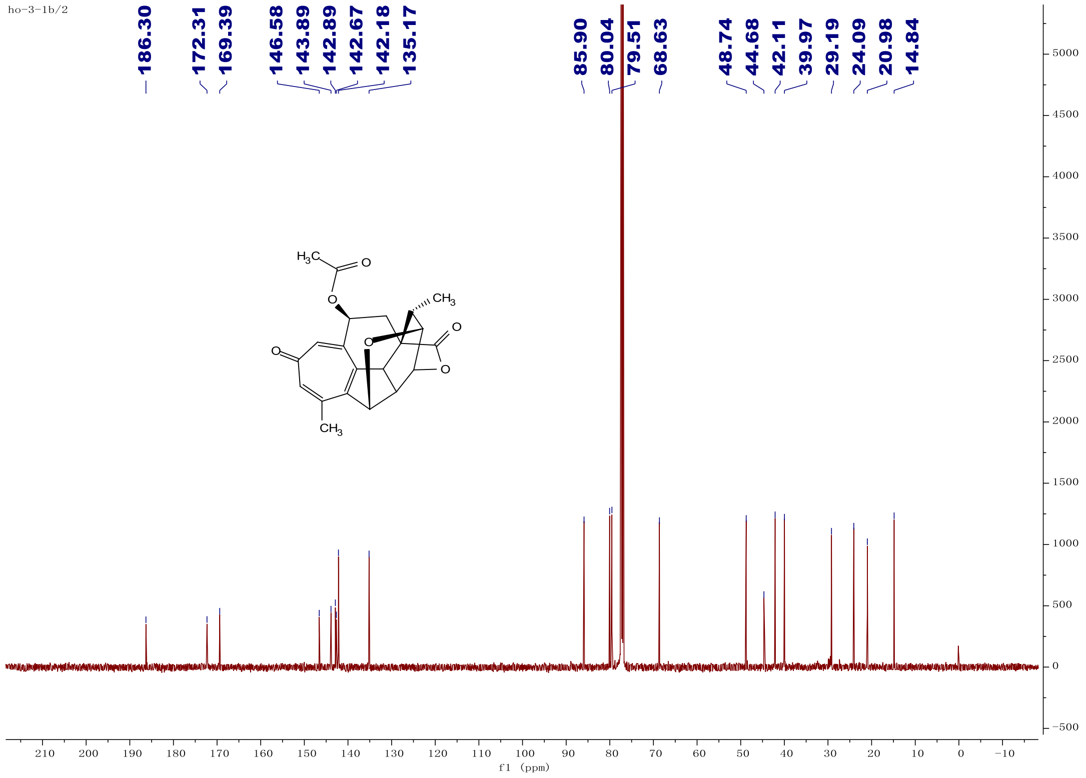

^{13}C-NMR spectrum (126 MHz, CDCl3) for 10a

Compound 10b

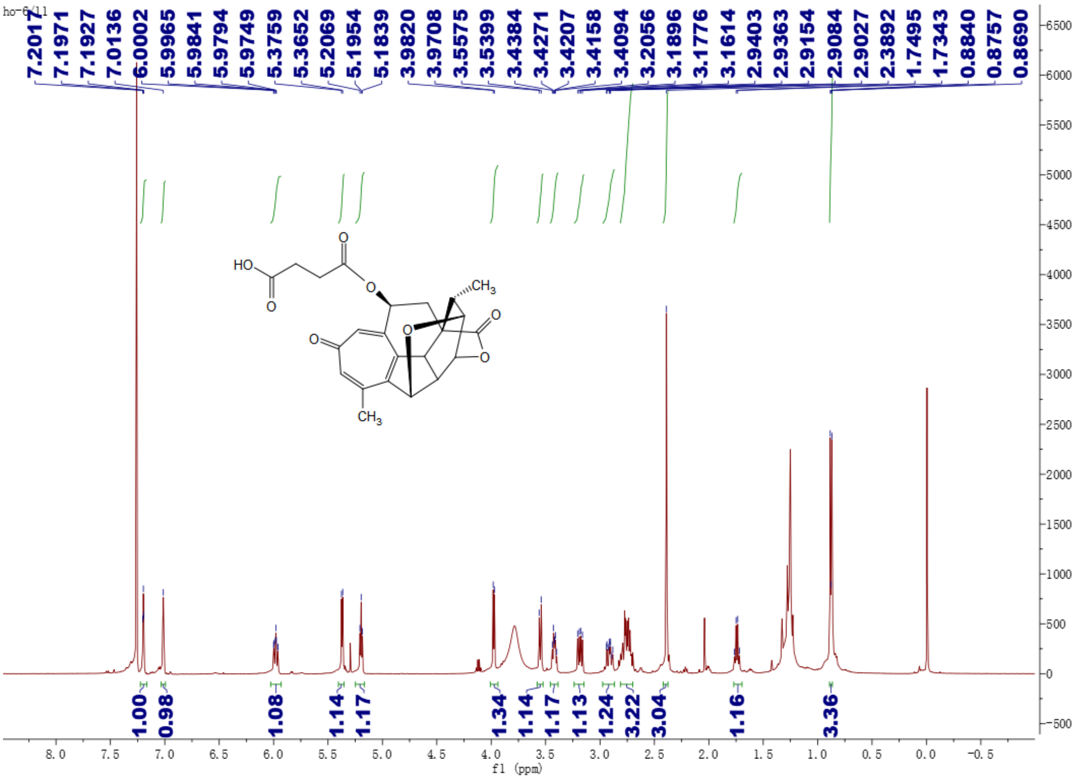

^{1}H-NMR spectrum (500 MHz, CDCl3) for 10b

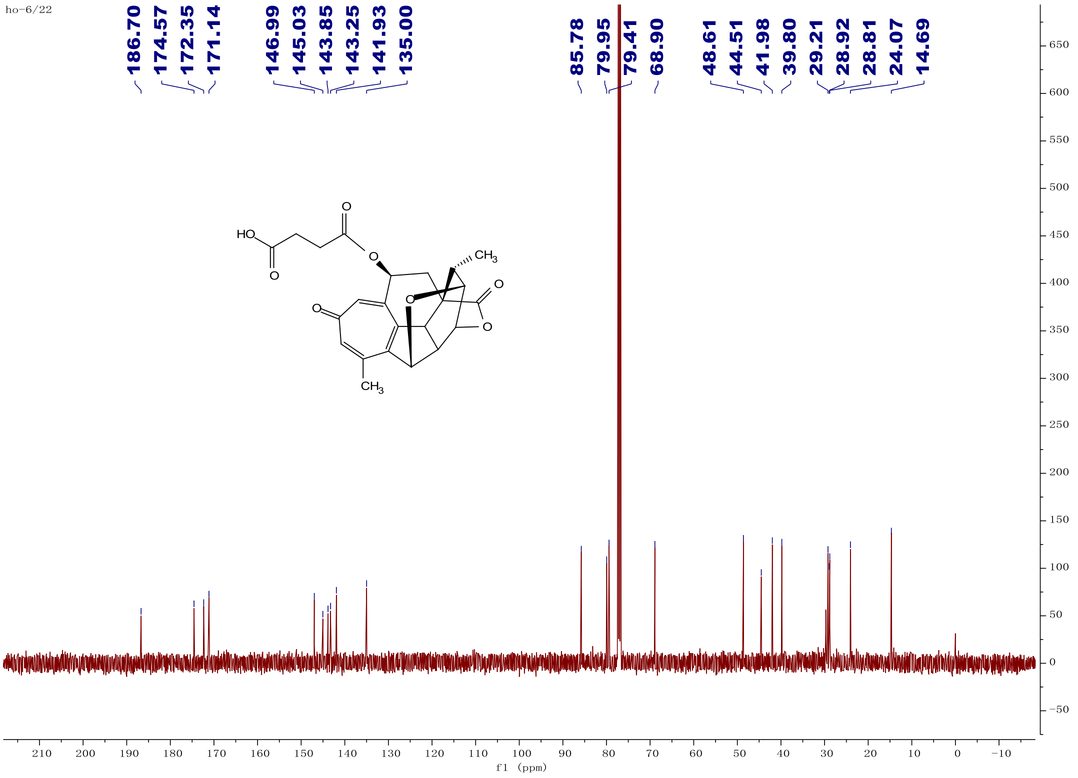

^{13}C-NMR spectrum (126 MHz, CDCl3) for 10b

Compound 10c

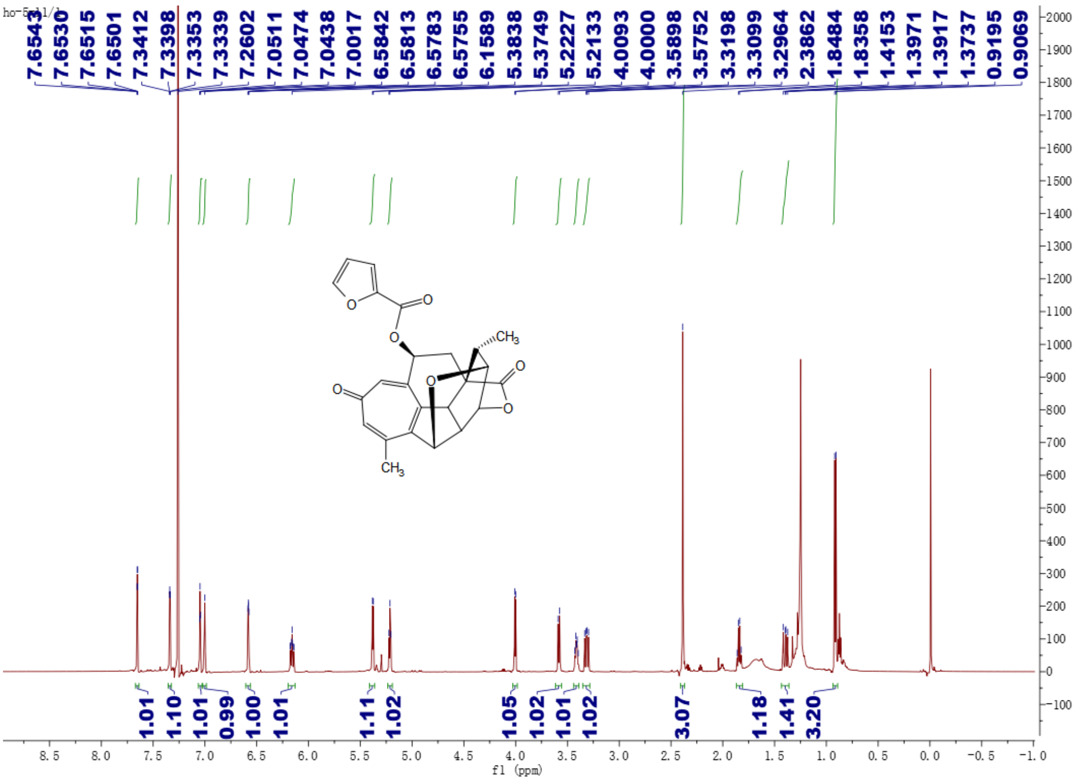

^{1}H-NMR spectrum (600 MHz, CDCl$_3$) for 10c

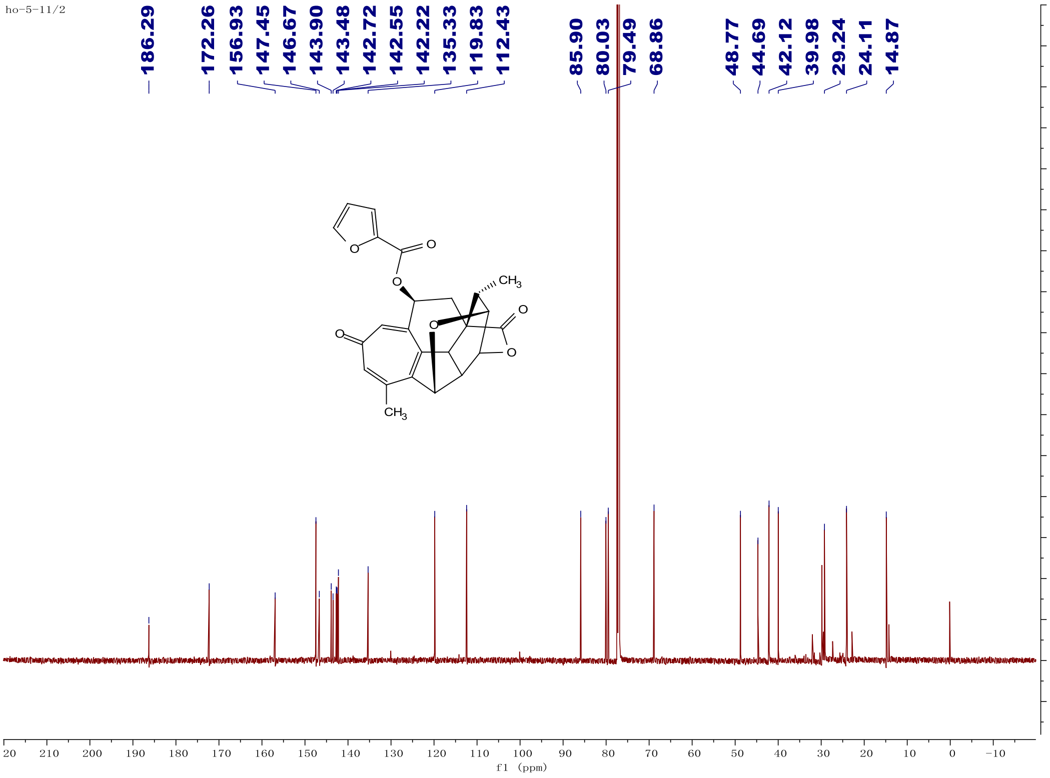

^{13}C-NMR spectrum (151 MHz, CDCl$_3$) for 10c

Compound 10d

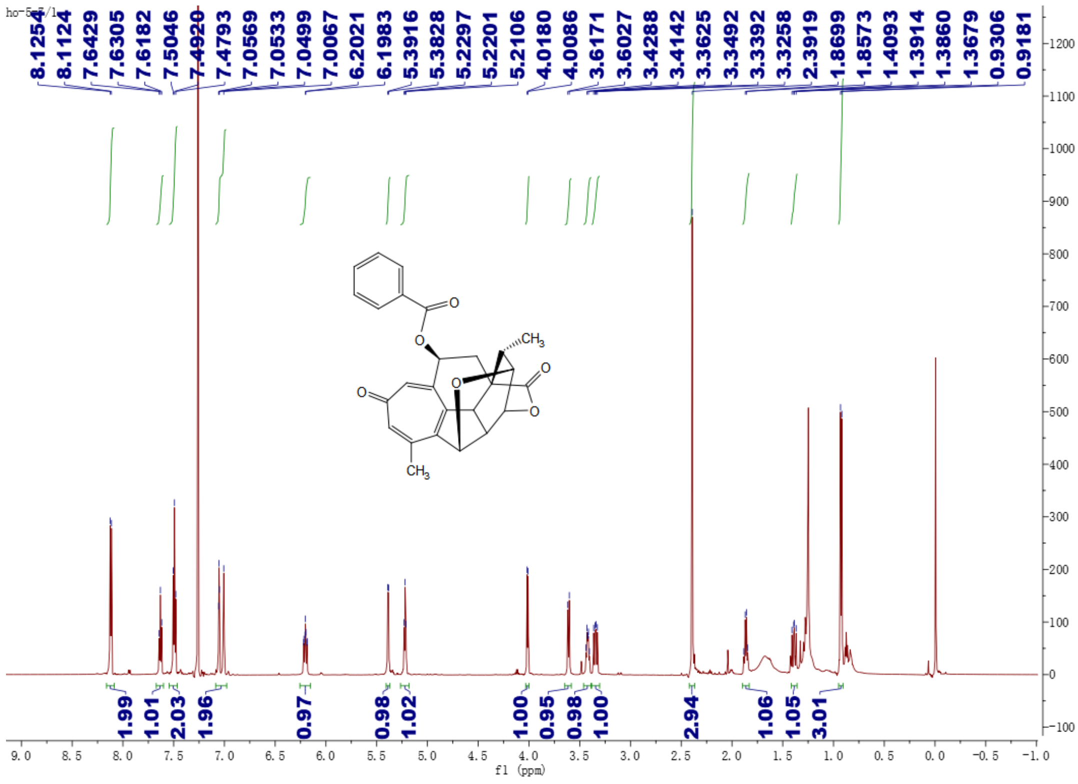

^{1}H-NMR spectrum (600 MHz, CDCl$_3$) for 10d

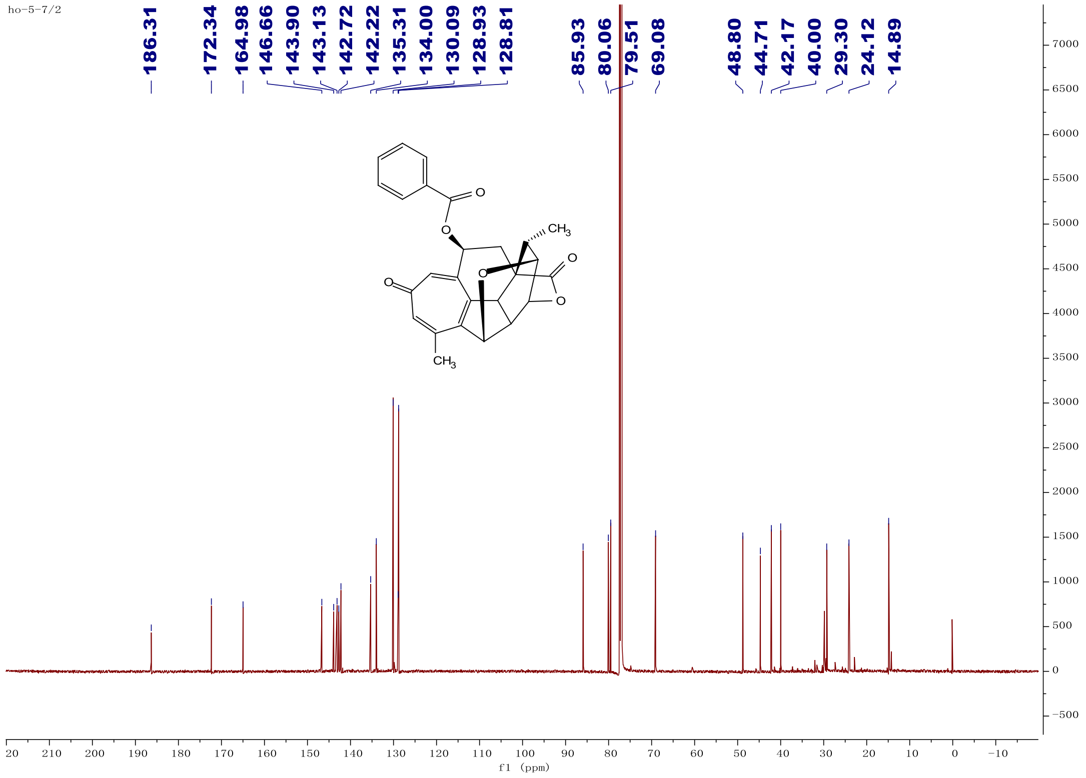

^{13}C-NMR spectrum (151 MHz, CDCl$_3$) for 10d

Compound 10e

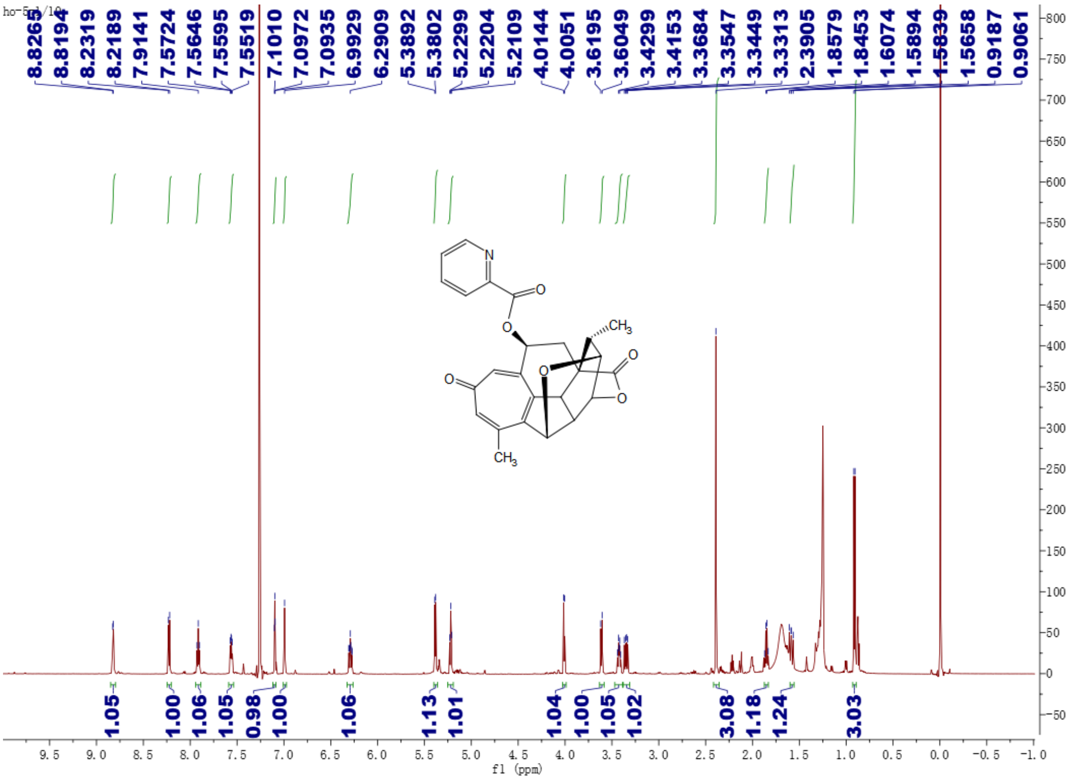

^{1}H-NMR spectrum (600 MHz, $CDCl_3$) for 10e

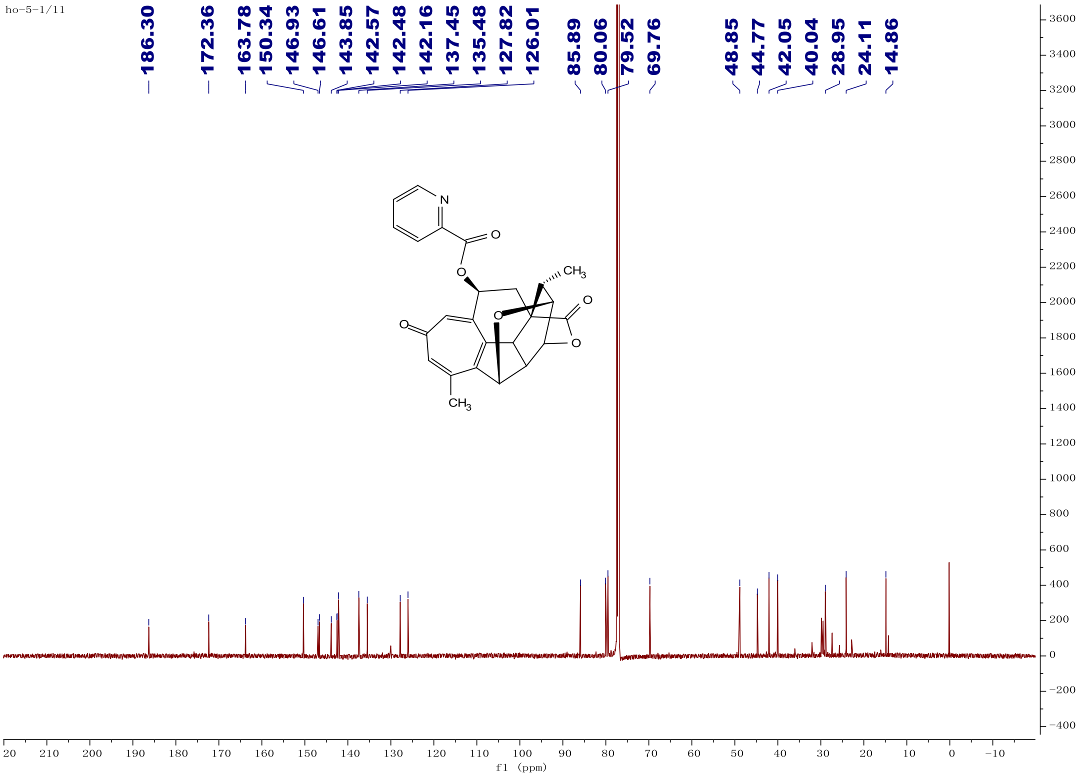

^{13}C-NMR spectrum (151 MHz, $CDCl_3$) for 10e

Compound 10f

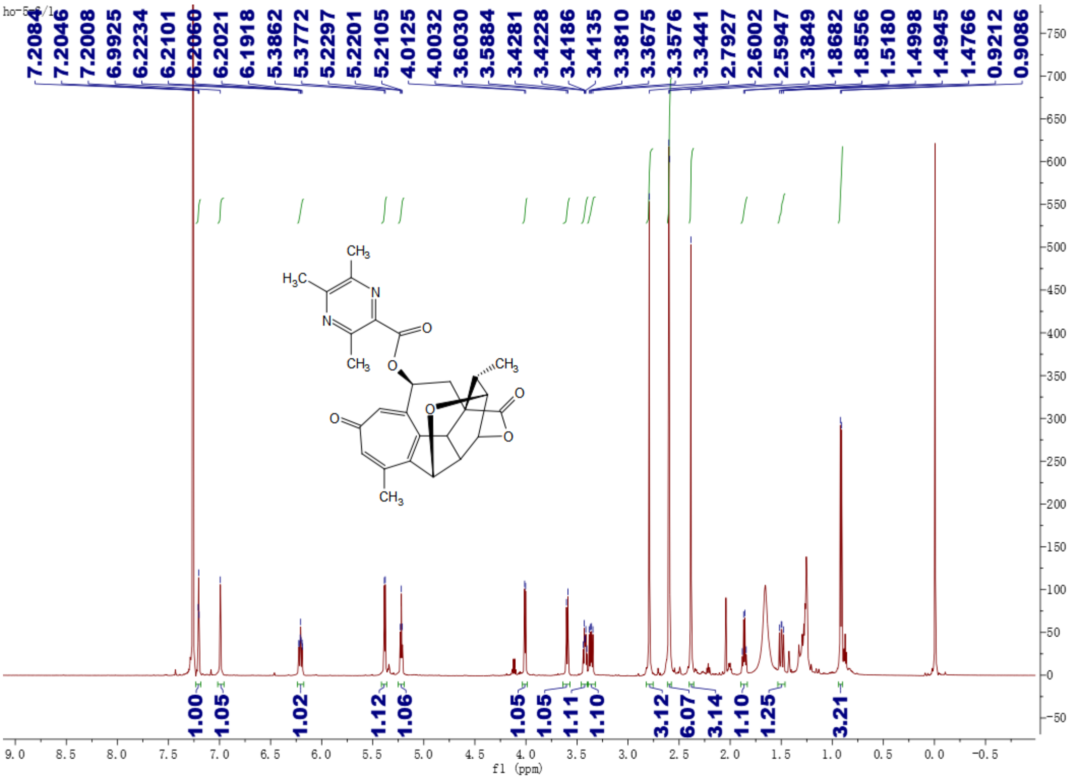

^{1}H-NMR spectrum (600 MHz, CDCl3) for 10f

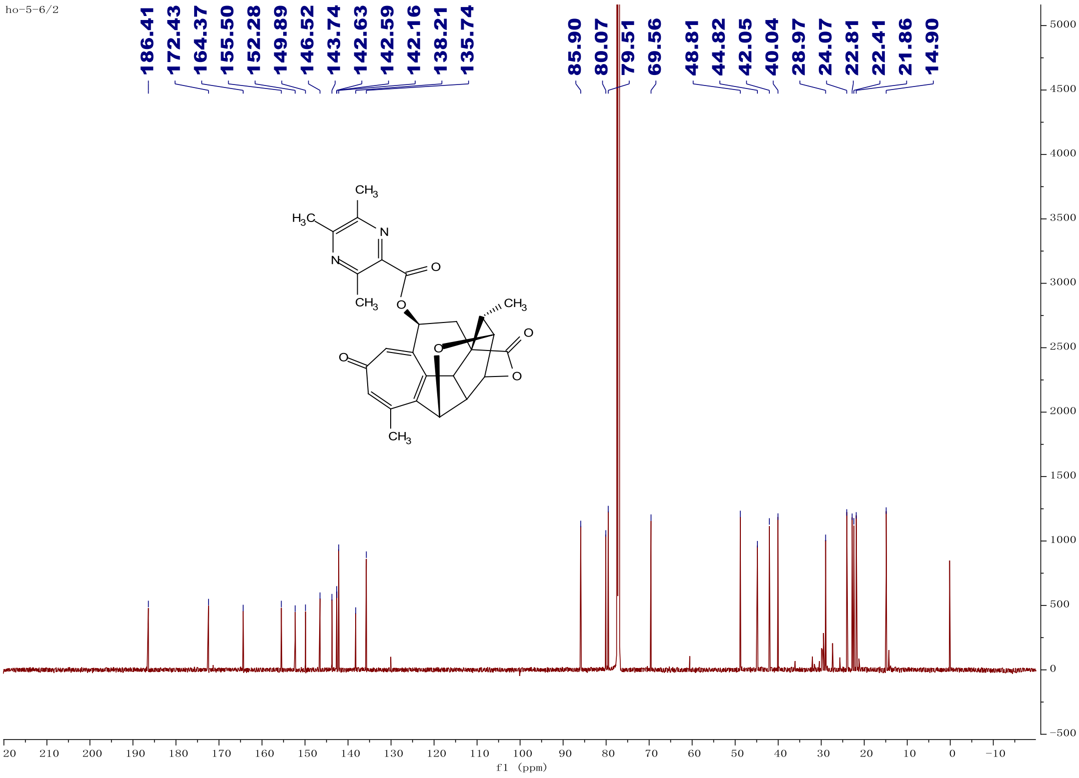

^{13}C-NMR spectrum (151 MHz, CDCl3) for 10f

Compound 11

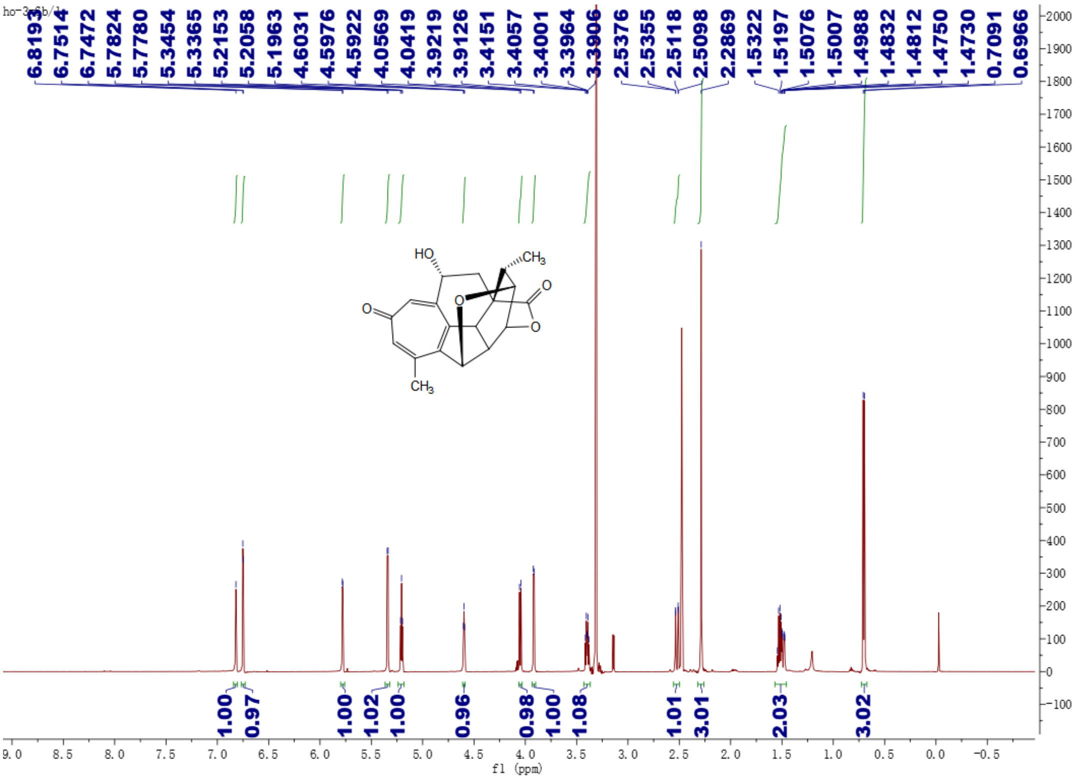

^{1}H-NMR spectrum (600 MHz, MeOD) for 11

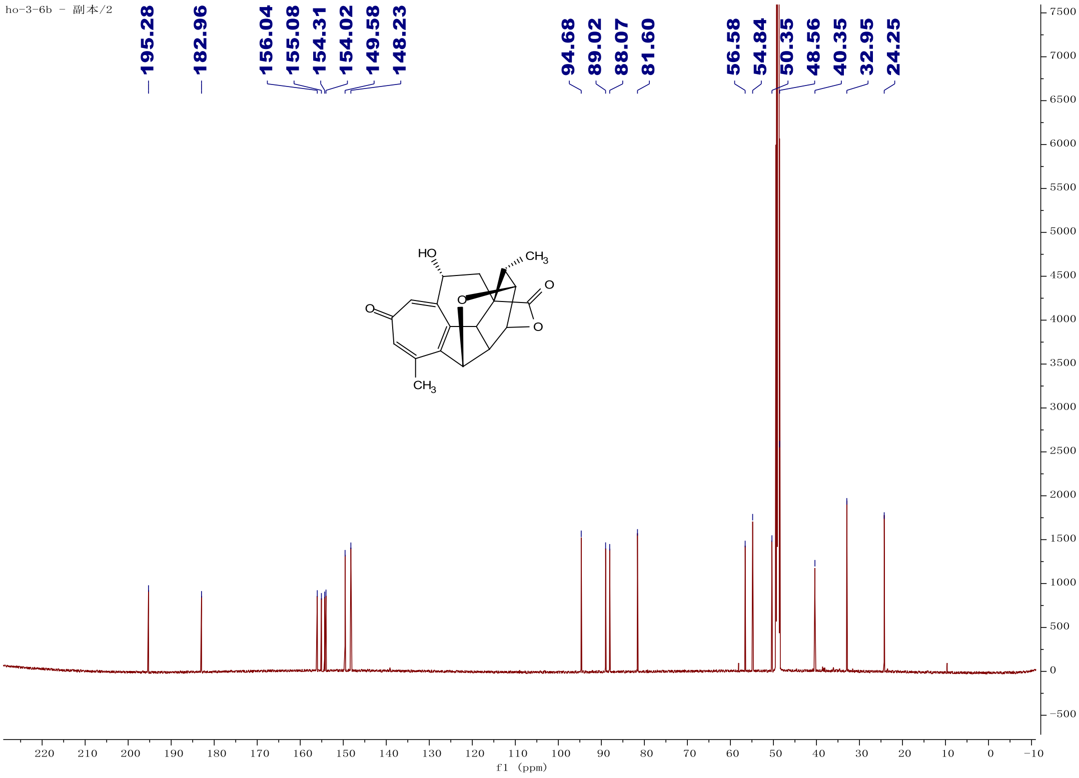

^{13}C-NMR spectrum (151 MHz, MeOD) for 11

Compound 12

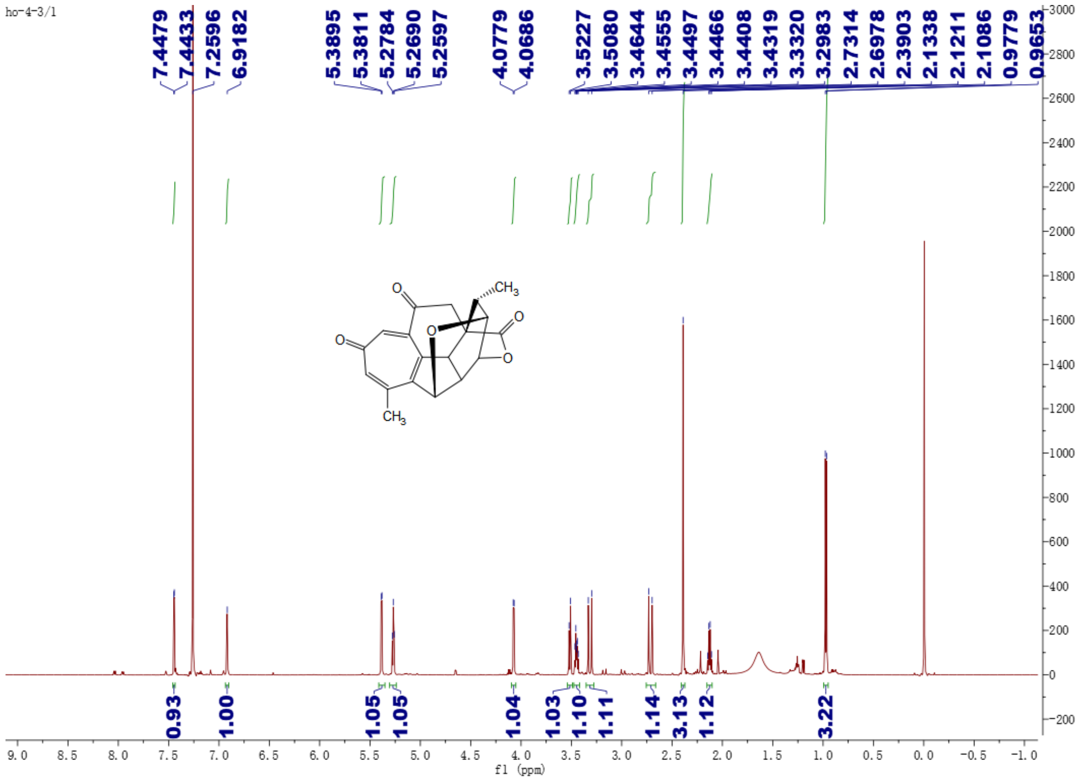

^{1}H-NMR spectrum (600 MHz, $CDCl_3$) for 12

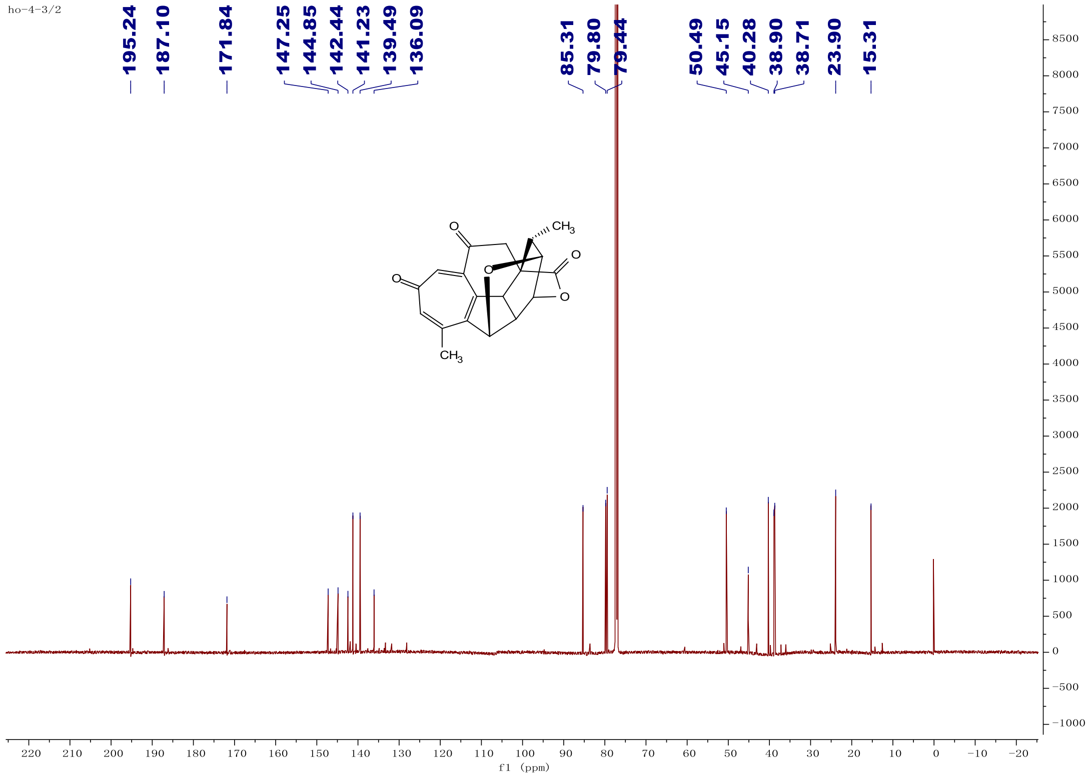

^{13}C-NMR spectrum (151 MHz, $CDCl_3$) for 12

References

1. Demidov, M.R.; Lapshina, M.Yu.; Osipov, D.V.; Osyanin, V.A.; Klimochkin, Y.N. Oxidative Rearrangement of 4*H*-Chromenes to 2-Aroylbenzofurans in the Presence of Selenium Dioxide. *Chem Heterocycl Comp* **2017**, 53, 1053–1056, doi:10.1007/s10593-017-2169-7.
2. Morita, S.; Yoshimura, T.; Matsuo, J. Intramolecular Büchner Reaction and Oxidative Aromatization with SeO_2 or O_2. *Chem. Pharm. Bull.* **2019**, 67, 729–732, doi:10.1248/cpb.c19-00243.
3. Karimi, S.; Ma, S.; Ramig, K.; Greer, E.M.; Szalda, D.J.; Subramaniam, G. Oxidative Ring-Contraction of 3*H*-1-Benzazepines to Quinoline Derivatives. *Tetrahedron Letters* **2015**, 56, 6886–6889, doi:10.1016/j.tetlet.2015.10.094.
4. Maier, W.F., Roth, W., Thies, I., Schleyer, P.V.R. Hydrogenolysis, iv. gas phase decarboxylation of carboxylic acids. *Chem. Ber.* **1982**, 115, 808-812, doi:10.1002/cber.19821150245
